# Introgression Threatens the Genetic Diversity of *Quercus austrocochinchinensis* (Fagaceae), an Endangered Oak: A Case Inferred by Molecular Markers

**DOI:** 10.3389/fpls.2017.00229

**Published:** 2017-02-21

**Authors:** Miao An, Min Deng, Si-Si Zheng, Xiao-Long Jiang, Yi-Gang Song

**Affiliations:** ^1^Shanghai Key Laboratory of Plant Functional Genomics and Resources, Shanghai Chenshan Botanical GardenShanghai, China; ^2^Shanghai Chenshan Plant Science Research Center, Chinese Academy of SciencesShanghai, China

**Keywords:** *Quercus* subgenus *Cyclobalanopsis*, AFLP, SSR, introgression, endangered species

## Abstract

Natural introgression can cause negative effects where rare species experience genetic assimilation and invade by their abundant congeners. *Quercus austrocochinchinensis* and *Q. kerrii* (subgenus *Cyclobalanopsis*) are a pair of closely related species in the Indo-China area. Morphological intermediates of the two species have been reported in this region. In this study, we used AFLP, SSR and two key leaf morphological diagnostic traits to study the two *Q*. *austrocochinchinensis* populations, two pure *Q. kerrii* and two putative hybrid populations in China. Rates of individual admixture were examined using the Bayesian clustering programs STRUCTURE and NewHybrids, with no a priori species assignment. In total, we obtained 151 SSR alleles and 781 polymorphic loci of AFLP markers. Population differentiation inferred by SSR and AFLP was incoherent with recognized species boundaries. Bayesian admixture analyses and principal coordinate analysis identified more hybrids and backcrossed individuals than morphological intermediates in the populations. SSR inferred a wide genetic assimilation in *Q. austrocochinchinensis*, except for subpopulation D2 in the core area of Xi-Shuang-Ban-Na Nature Reserve (XSBN). However, AFLP recognized more *Q. austrocochinchinensis* purebreds than SSR. Analysis using NewHybrids on AFLP data indicated that these hybridized individuals were few F_2_ and predominantly backcrosses with both parental species. All these evidences indicate the formation of a hybrid swarm at XSBN where the two species co-exist. Both AFLP and SSR recognized that the core protected area of XSBN (D2) has a high percentage of *Q. austrocochinchinensis* purebreds and a unique germplasm. The Hainan population and the other subpopulations of XSBN of the species might have lost their genetic integrity. Our results revealed a clear genetic differentiation in the populations and subpopulations of *Q. austrocochinchinensis* and ongoing introgression between *Q. austrocochinchinensis* and *Q. kerrii* at the disturbed contact areas. Combining the results from genetic and morphological analyses, the conservation of subpopulation D2 should be prioritized. Conservation and restoration of the integrity of tropical ravine rainforest is an important long-term goal for the successful conservation of *Q. austrocochinchinensis*. The fine-scale landscape might play an essential role in shaping the spatial patterns of hybridization. Further studies are needed to evaluate these patterns and dynamics.

## Introduction

Natural hybridization is a frequent phenomenon in plants, occurring in 25% of extant species (Mallet, 2005; Whitney et al., 2010). The F_1_ hybrids without reproductive barriers can bridge the gene flow between the two parental species by facilitating further backcrossing to the parental species; this process leads to introgression, which is an important evolutionary process (Arnold, 1992; Barton, 2001). By interspecific genetic exchange, introgression increases the genetic diversity of one or both parental species and can lead to novel adaptations and speciation events (Grant, 1981; Rieseberg, 1995, 1997; Mallet, 2007). However, hybridization and introgression events can also have harmful effects on the progenitor's fate. An endemic or rare species may go extinct when it undergoes introgression with common congeners or a more reproductively successful prevalent species (Rieseberg, 1995; Levin et al., 1996; Rhymer and Simberloff, 1996; Lepais et al., 2009). By repeated backcrossing, ancestral alleles of rare species become diluted after a certain number of generations (Briggs and Walters, 1997).

The genus *Quercus* s.l. contains ~400 to 600 species (Govaerts and Frodin, 1998) and can be divided into the subgenera *Quercus* and *Cyclobalanopsis*, based on whether the cupule is imbricate-scaled or lamellate. The subgenus *Cyclobalanopsis* is one of the dominant tree taxa in evergreen broad-leaf forests (EBLFs) of eastern and southeastern Asia, with ~90 to 122 species (Govaerts and Frodin, 1998; Deng, 2007). Natural introgression is common in oaks (Valbuena-Carabaña et al., 2005; Curtu et al., 2007; Burgarella et al., 2009; Salvini et al., 2009; Ortego and Bonal, 2010; Moran et al., 2012). Renowned as “worst case scenario for the biological species concepts” (Coyne and Orr, 2004) due to apparent local interspecific gene flow (Burger, 1975; Whittemore and Schaal, 1991; Lexer et al., 2006), widespread oak species of the subgenus *Quercus*, nonetheless, exhibit genetic coherence across a broad geographic range (Muir et al., 2000; Hipp and Weber, 2008; Cavender-Bares and Pahlich, 2009). However, most of these studies were conducted on species of the subgenus *Quercus*.

Although trees of the subgenus *Cyclobalanopsis* are the keystone elements in EBLFs of mainland Asia, studies on hybridization and introgression among the species of this subgenus are rather rare. Only two sympatric species (*Q. sessilifolia* and *Q. acuta*) distributed in Korea and Japan were investigated and revealed the introgression between the two species (Tamaki and Okada, 2014). Indo-China is the diversification center for the subgenus *Cyclobalanopsis*, with about 70 species occurring in EBLFs in this region (Lou and Zhou, 2001). Of these, one-third are endemic and rare species, and a large number of them have sympatric distributions, but maintain their prominent ecological niche and morphological variation. So far, no studies have applied genotyping methods to test the existence of gene flow among the different species of the subgenus *Cyclobalanopsis* in Indo-China. *Quercus kerrii* and *Q. austrocochinchinensis* is a pair of species closely genetically related to the subgenus Cyclobalanopsis (Deng et al., 2013). *Q. kerrii* is widespread common species in open slopes of EBLFs in Indo-China, while the distribution of *Q. austrocochinchinensis* is rather restricted, with only four known sites, of which two are in China, and other two are located in Northern Vietnam and Northern Thailand respectively (Huang et al., 1999; Phengklai, 2006).

We have previously described the morphological intermediates *Q. austrocochinchinensis* and *Q. kerrii* using leaf morphological traits, indicating that the two species can form hybrids (Song et al., 2015). Intermediate morphology has been widely used to reveal the status of hybrids in former studies of plant hybridization (Kleinschmit et al., 1995; Craft et al., 2002; Kremer et al., 2002). However, morphological diagnostic traits have limited power to accurately identify the hybrids and pure parental species (López-Caamal and Tovar-Sánchez, 2014). Compared to morphologic methods, DNA markers are more reliable and powerful tools compared (Harrison, 1993) and can also precisely predict the ancestral states in later generation hybrids (Pritchard et al., 2000; Falush et al., 2003; Evanno et al., 2005). Preserving the genetic distinction of endangered species is critical for their conservation and by using molecular approaches, it is possible to select out non- or less-hybridized subpopulations from a hybrid zone to use in *ex-situ* conservation.

In this follow-up study, we aim to (1) investigate whether and to what extent introgression exists between *Q. austrocochinchinensis* and *Q. kerrii*; (2) discuss the genetic extinction risk of the rare oak species *Q. austrocochinchinensis* and its possible conservation management; (3) compare the results of different approaches (morphological traits, AFLP, and SSR), and discuss the diagnostic power in distinguishing hybrids.

## Materials and methods

### Ethics statement

Sampling of endangered oak species *Quercus austrocochinchinensis* and *Q. kerrii* was granted and supported by National Forestry Bureau of China and Local National Nature Reserves.

### Population sampling and species identification

**Figure 1:**
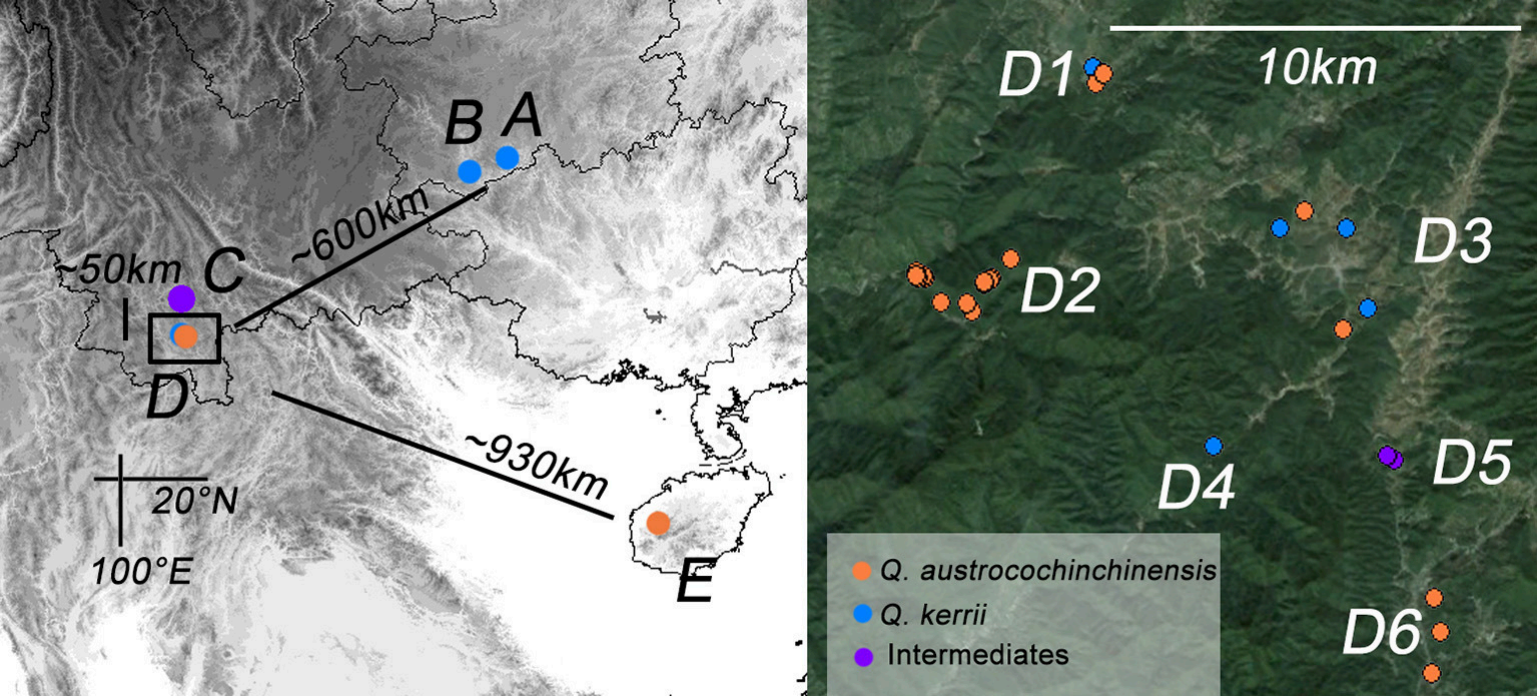
**Geographic distribution of the study populations of ***Quercus kerrii*** and ***Q. austrocochinchinensis*****. Population A and B are pure *Q. kerrii* populations. C is a putative hybrid population without co-occurrence of any *Q. austrocochinchinensis* trees. D1–D9 are located in the contact zone. E is a population of *Q. austrocochinchinensis* that has been reported previously in a morphological study as a purebred population (Song et al., 2015). Individuals were colored according to parental species or intermediates (orange, blue, or purple) pre-identified by morphological features.

In total, 57 and 36 individuals with typical traits of *Q. austrocochinchinensis* and *Q. kerrii*, respectively, were used and 15 morphological intermediates were included in this study. The 108 individuals were sampled in five populations, A–E (Figure 1 and Table 1). According to a previous investigation (Song et al., 2015), *Q. austrocochinchinensis* trees can only be found in populations D and E. Of these, E was considered as a pure *Q. austrocochinchinensis* population (Song et al., 2015) and D is located in the contact zone which contains both *Q. kerrii* and *Q. austrocochinchinensis* trees. Six sub-populations, D1–D6, were sampled within D population regions. Two putative *Q. kerrii* purebred populations were sampled from populations A and B. Population C is a putative hybrid population with morphological intermediates, but trees with typical traits of *Q. austrocochinchinensis* cannot be found in or close to region C.

**Table 1:** **Sampling information of the study populations**.

**Site ID**	**Population type**	**Location**	**N(a)**	**N(k)**	**N(i)**	**Long (E)**	**Lat (N)**	**Elev (m)**
A	Pure site	Luo-dian, Guizhou	0	11	0	106°40′	25°15′	526
B	Pure site	Heng-xian, Guizhou	0	17	0	105°53′	24°59′	459
C^*^	Intermediate	Si-mao, Yunnan	0	0	10	100°50′	22°50′	1077
D1	Mixed site	XSBN, Yunnan	2	2	0	100°48′	22°05′	919
D3	Mixed site	XSBN, Yunnan	14	4	0	100°52′	22°19′	1030
D2	Pure site	XSBN, Yunnan	25	0	0	100°46′	22°19′	867
D6	Pure site	XSBN, Yunnan	7	0	0	100°53′	22°14′	867
D4	Pure site	XSBN, Yunnan	0	2	0	100°50′	22°17′	1036
D5	Intermediate	XSBN, Yunnan	0	0	5	100°53′	22°17′	936
E	Pure site	BWL, Hainan	9	0	0	109°05′	19°07′	247

*XSBN, Xi-Shuang-Ban-Na National Nature Reserve; BWL, Ba-Wang-Ling Nature Reserve; N (a/k/i): sampling number of Q. austrocochinchinensis (a), Q. kerrii (k) and morphological intermediates (i). Long, Longitude; Lat, Latitude; Elev, Elevation*.

Prior to the experiment, all individuals and their voucher specimens were carefully inspected and identified based on key morphological diagnostic traits, e.g., shape of cupule and trichomes on leaf abaxial surface. The results were used as species information for the studied samples in later analyses. Leaf tissues used for DNA extractions were collected from each individual and dried instantly using silica gel. All vouchers specimens of each tree were stored at the herbarium of the Shanghai Chenshan Botanical Garden (CSH).

### Measurement of leaf morphological traits

**Figure 2:**
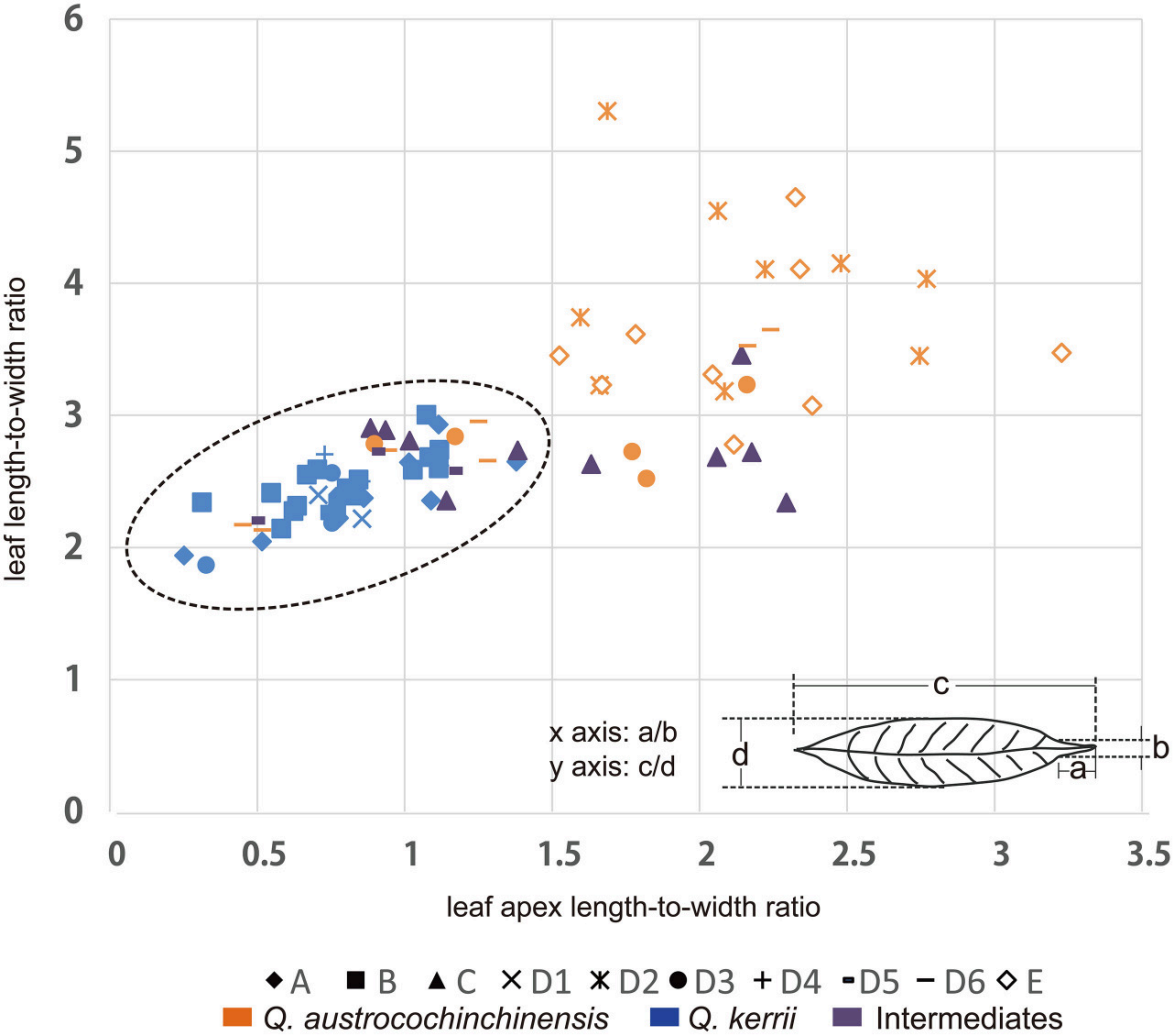
**Distributions of two leaf morphological measures**. Graph displaying the relationship between the two morphological features that were measured in this study, where the leaf apex length-to-width ratio is on the x-axis and the leaf length-to-width ratio is on the y-axis. “♦” represents Population A; “■” represents Population B; “▴” represents Population C; “♢” represents Population E; the remaining symbols belong to Population D, of which “×” represents sub-population D1; “
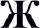
” represents sub-population D2; “•” represents sub-population D3; “+” represents subpopulation D4; “
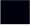
” represents subpopulation D5; “
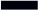
” represents sub-population D6.

In a previous study, we found that the two macro-morphological features leaf apex shape and leaf length-to-width ratio are the key diagnostic features that can be applied to identify *Q. kerrii* and *Q. austrocochinchinensis* and even to assign their hybrids (Song et al., 2015). To link the genetic pattern to the morphological features, we used ratios instead of absolute lengths to measure leaf morphological traits for the two parental species and the intermediates. The two ratios were leaf length-to-width ratio and leaf apex length-to-width ratio. The measuring method of the two ratios is shown in Figure 2. At least five leaves were measured from each voucher specimen to compute an average for the drawing of the scatter plot.

### SSR and AFLP analysis

Total genomic DNA for each sampled individual was extracted from the silica gel-dried leaf tissue using the modified CTAB method (Huang et al., 2000). The DNA quality was checked by loading DNA on a 1.0% agarose gel, and the DNA concentration of each sample was measured using a TBS-380 Fluorometer (Turner BioSystems Inc., Sunnyvale, CA). The samples were screened for 11 SSR markers. Of these loci, Qk15874, Qk17139, Qk17611, and Qk20944 have been developed for *Q. kerrii* from transcriptome data (An et al., 2016), QmC00693, QpZAG9, QpZAG16, QpZAG36, QpZAG110, CG371 were from the non-coding region (Steinkellner et al., 1997; Ueno et al., 2008; Tong et al., 2012), and CR627959 was predicted in the coding region of Cys-3-His zinc finger protein in nuclear genome (Ueno and Tsumura, 2008). All of these loci show a relatively high degree of transferability within the genus *Quercus* and an adequate degree of polymorphism of the studied taxa. For the 11 SSR primer pairs, 5′ end of forward primers were labeled with fluorescent dye tags (6FAM or HEX or ROX) (Sangon, Shanghai, China). The PCR reactions were performed in 20 μl reaction volume containing 10 μl TIANGEN PCR Master Mix (TIANGEN, Beijing, China), 0.3 μl/L of each primer (10 mM), and 20 ng genomic DNA; PCR reactions were performed as follows: 5 min initial denaturation at 94°C, 35 cycles of 40 s at 94°C, 30 s at 55°C, 1 min elongation at 72°C, and 7 min extension at 72°C. Finally, Gene-Scan-500 LIZ size standard (Applied Biosystem™) was added to all the samples before loading on an automated sequencer ABI 3730 (Applied Biosystem™). The final step was performed by a private professional commercial lab (Shanghai Majorbio Bio-pharm Technology Co., Ltd, Shanghai, China).

The AFLP method was performed following the protocol by Vos et al. (1995), with minor modifications. Briefly, 500 ng of genomic DNA were double-digested using 5 U of *EcoR*I and 2 U of *Mse*I (New England BioLabs). The digestion mixtures were incubated at 37°C for 3 h and the digested mixture was then incubated at 70°C for 10 min to denature the enzymes. Subsequently, 4 μL of digested DNA were added to 16 μL of ligation mix containing 2 U T4 DNA ligase (New England BioLabs), 5 pmol *EcoR*I, and 50 pmol *Mse*I adaptor. The mixture was incubated at 10°C for 14 h and then denatured at 70°C for 10 min. The ligated DNA samples were diluted 5-fold with double sterile water. Pre-selective amplification reactions were carried out using *Eco*RI-A (5′-GACTGCGTACCAATTCA-3′) and *Mse*I-C (5′-GATGAGTCCTGAGTAAC-3′) in a 50 μL volume containing 1.5 mmol/L MgCl_2_, 200 μmol/L of each dNTP, 1.25 μmol/L of each primer, and 0.6 U *r*Taq DNA polymerase (Takara Biotechnology, Dalian, China) under the following cycle: 3 min at 72°C, 30 cycles of 30 s denaturing at 94°C, 30 s annealing at 56°C, 1 min extension at 72°C, and a final extension for 5 min at 72°C. After a 1:20 dilution of pre-selective PCR products, seven primer combinations were performed in selective amplification (*EcoR*I-ACG/*Mse*I-CAC, *EcoR*I-AGG/*Mse*I-CAA, *EcoR*I-AGG/*Mse*I-CAC, *EcoR*I-AGG/*Mse*I-CAG, *EcoR*I-AGG/*Mse*I-CTA, *EcoR*I-AGG/*Mse*I-CTC, *EcoR*I-AGG/*Mse*I-CTT). The *Eco*RI primers were fluorescently labeled with 6-FAM. These primer pairs were chosen because they generated clear and fewer bands (thus decreasing the risk of fragment non-homology) with sufficient variability in preliminary tests. Selective PCRs were carried out in a 20 μL volume containing 2.5 mmol/L MgCl_2_, 200 μmol/L of each dNTP, 1.25 μmol/L of each primer, and 0.2 U of rTaq DNA polymerase (Takara Biotechnology, Dalian, China) and under the following cycle: 3 min at 94°C, 9 cycles of 40 s at 94°C, 30 s at 65–57°C touchdown (reducing the temperature at 1°C per cycle), 15 min at 72°C, 20 cycles of 40 s at 94°C, 30 s at 56°C, 1.5 min at 72°C, and a final extension for 7 min at 60°C. The PCR products were 10-fold diluted and mixed with Gene-Scan-500 LIZ size standard (Applied Biosystem™); products from each primer combination were loaded separately on an automated sequencer ABI 3730 (Applied Biosystem™) by the same commercial service provider mentioned above.

Raw data of SSR and AFLP samples were collected and analyzed using GeneMarker®v2.2.0. The samples with low quality of size calibrations or peaks were excluded from allele calling. The allele sizes of SSR were read manually and MicroChecker v 2.2.3 (Van Oosterhout et al., 2004) was used to check for potential errors. For AFLP, the variable fragments in the size range 50–500 base pairs (bp) were manually scored as present (1) or absent (0). We only considered fragments with similar fluorescence profile and intensities across the samples to maximize the probability of homology.

### Genetic diversity analysis

A set of statistical tests on SSRs, including allelic richness (Na), allele frequency distribution, Shannon's Information Index (*I*), Observed and Expected Heterozygosity (*H*_O_ and *H*_E_), were performed by GenAlEx version 6.5 (Peakall and Smouse, 2012), using all the individuals of *Q. kerrii* and *Q. austrocochinchinensis*. In addition, Unbiased Expected Heterozygosity (u*H*_E_) was estimated as (2N/(2N-1)) * *H*_E_. Fixation Index (*F*_IS_), or inbreeding coefficient was estimated as (*H*_E_–*H*_O_)/*H*_E_ (Nei and Li, 1979). We used GenePop v 4.2 (Rousset, 2008) to test the departure from Hardy-Weinberg equilibrium (HWE) (heterozygote deficiency or excess) for each locus of the 11 SSR loci, and to test for homogeneity of alleles distributions between species. We also counted the number of private alleles for each species.

For AFLP, percentage of polymorphic loci, unbiased estimates of genetic diversity (*H*_j_, analogous to *H*_E_), and differentiation statistics were calculated using the AFLP-SURV v. 1.0 software (Vekemans et al., 2002). With this software, allelic frequencies at AFLP loci were calculated from the observed frequencies of fragments using the Bayesian approach proposed by Zhivotovsky (1999) for diploid species. A non-uniform prior distribution of allelic frequencies was assumed with its parameters derived from the observed distribution of fragment frequencies among loci. These allelic frequencies were used as the input for the analysis of genetic diversity within and between samples following the method described in Lynch and Milligan (1994).

### Genetic and phenotypic differentiation

The comparison of *F*_ST_ and *Q*_ST_ provides a basis to distinguish neutral from adaptive divergence (Leinonen et al., 2013). To investigate genetic differentiation between the two species and among populations, *F*_ST_ values were measured on AFLP and SSR data using AFLP-SURV v. 1.0 (Vekemans et al., 2002) and GenePop v. 4.2 (Rousset, 2008), respectively. The parameter *Q*_ST_ estimates the among-population proportion of the total additive genetic variance of a genetic quantitative trait. If *Q*_ST_ = *F*_ST_, trait divergence among populations could have been driven only by genetic drift. If *Q*_ST_ > *F*_ST_, the populations are likely to have been caused by directional selection, and if *Q*_ST_ < *F*_ST_, there is evidence for uniform selection or stabilizing selection across the populations. Because this is not possible for the breeding designs for this study, we used *P*_ST_, a proxy for *Q*_ST_, to compare with *F*_*ST*_ (Leinonen et al., 2013). In this study, *P*_ST_ was calculated between all the *Q. austrocochinchinensis* and *Q. kerrii* individuals, using the equation of $\sigma_{\text{GB}}^{2}$/($\sigma_{\text{GB}}^{2}+$2$\sigma_{\text{GW}}^{2}$), where $\sigma_{\text{GW}}^{2}$ and $\sigma_{\text{GB}}^{2}$ are within- and among-population components of variance. Putative hybrids were excluded from *F*_ST_ and *P*_ST_ estimation.

In addition, *F*_ST_ for each locus was also estimated. For AFLP, allele frequencies of *Q. austrocochinchinensis* and *Q. kerrii* were calculated separately. We calculated *F*_ST_ values between 57 for *Q. austrocochinchinensis* and 36 for *Q. kerrii* individuals for all polymorphic loci via the formula *F*_ST_ = 1-*H*_S_/*H*_T_ (Nei, 1973). The variable *H*_S_ represents average within-population heterozygosity and *H*_T_ represents expected heterozygosity for the total population; *H*_S_ and *H*_T_ were calculated using the following formula: *H*_S_ = 1/2(2p_1_q_1_+2p_2_q_2_) and *H*_T_ = 1/2(p_1_+p_2_)*(q_1_+q_2_), with q = 1-p. For SSR markers, *F*_ST_ for each loci was calculated using GenePop v. 4.2 (Rousset, 2008). Finally, the frequency distribution of *F*_ST_ values for both markers were plotted in a histogram.

To detect the outlier loci under selection of an AFLP dataset (781 loci), program BayeScan v. 2.1 (Foll and Gaggiotti, 2008; Fischer et al., 2011) was used to identify candidate loci under natural selection across all 10 populations. A threshold value for determining loci under selection was evaluated in accordance with Jeffreys (1961) interpretation, which is a logarithmic scale for model choice as follows: log_10_PO > 0.5 (substantial), log_10_ PO > 1.0 (strong), log_10_ PO > 1.5 (very strong), and log_10_PO > 2.0 (decisive support for accepting a model) (Fischer et al., 2011). We employed a threshold of log_10_ PO > 2.0 for the rejection of the null hypothesis in each of the conducted tests. BayeScan analysis was conducted with a burn-in of 50,000 iterations, a thinning interval of 50, and a sample size of 5,000. The number of pilot runs was kept at 20, with a length of 50,000 each. The SSR loci were not used to calculate outliers because of their limited number.

### Population cluster analysis

To cluster individuals into genetically distinct groups, a Bayesian clustering approach was employed using STRUCTURE v. 2.3.4 (Pritchard et al., 2000; Falush et al., 2003), without consideration of sampling information. We adopted the admixture model with correlated allele frequencies (Lepais et al., 2009; Zalapa et al., 2009). No prior knowledge of the species was included in the analyzed data sets. To determine the optimal number of groups (K), we ran STRUCTURE, with K varying from 1 to 10 and with 10 runs for each K value. The Δ*K* was calculated using the mean log-likelihood for each K according to Evanno et al. (2005). For SSR and AFLP data sets, each run was performed for 100,000 Markov Chain Monte Carlo (MCMC) repetitions with a burn-in period of 50,000.

The admixture coefficient (*q*-value) generated from STRUCTURE was used to classify individuals into purebred and hybrids, using a threshold *q*-value of = 0.1, where samples with *q*-values < 0.1 or > 0.9 were classified as purebred and those with *q*-values between 0.1 and 0.9 as hybrids, including F_1_ and backcrosses (Vähä and Primmer, 2006; Lepais et al., 2009). The F_1_ hybrids result in *q*-values = 0.5, but the coefficient of backcrosses would be biased toward one of the parental species and produce *q*-values between 0.5 and 0.9 (Lepais et al., 2009). Taking errors into consideration, individuals with 0.6 < *q*-values < 0.9 were recognized as backcrosses.

As the SSR loci were tested to violate the HWE assumption (see result), we also used the program InStruct (Gao et al., 2007), an alternative software of STRUCTURE, to ensure that the results obtained from STRUCTURE were reliable. The HWE within loci for co-dominant markers is not compulsory for the model in InStruct. The optimal K (number of clusters) value was selected according to Deviance Information Criteria (DIC) and is presented in the result section. Mode 2 was selected to run 50,000 MCMC repetitions with 10,000 burn-in periods.

The NewHybrids software v. 1.1 beta, employing a Bayesian analysis (Anderson and Thompson, 2002; Anderson, 2008), was used as a second method to confirm the presence of hybrids in our dataset. It calculates the posterior probability that sampled individuals fall into one set of hybrid categories (Parent K, Parent A, F_1_, F_2_, BC to Parent K; BC to Parent A, thus covering parents and two generations of offspring). All the 11 SSR loci were analyzed using this software. For AFLP data, NewHybrids will assign the individuals more accurately, using the loci with high differentiation at interspecies level. Therefore, we filtered the AFLP loci according to *F*_ST_. We applied three approaches to compare the results obtained by NewHybrids, using 100 loci with highest *F*_ST_ value at interspecies level, 279 loci (*F*_*ST*_ ≥ 0.1), and 450 AFLP loci (*F*_ST_ ≥ 0.03), respectively, for analysis. A burn-in period of 7,500 MCMC repetitions was defined and 10,000 iteration were run thereafter.

Principal coordinate analysis (PCoA) aims to visualize similarities or dissimilarities of individual data based on a distance matrix. This method is also an alternative algorithm without any assumptions about the population genetic model. For AFLP and SSR data, the pairwise Euclidian distance matrix was constructed, and the first two principal co-ordinates were visualized by GenAlEx v. 6.5 (Peakall and Smouse, 2012).

## Results

### Morphological analysis

Morphological measurements showed that the two species could be distinguished using a combination of two ratio values, which were leaf length-to-width ratio (LR) and leaf apex length-to-width ratio (LAR) (Figure 2). We found that all *Q. kerrii* individuals were grouped together, with an average of LR = 2.45 (1.88–3.02) and LAR = 0.78 (0.23–1.36). Of the sampled *Q. austrocochinchinensis* individuals, only the samples from D2 and E were totally separated from *Q. kerrii* samples. They had larger LR and LAR values compared to other populations, with an average of LR = 3.71 (2.79–5.32) and LAR = 2.06 (1.50–3.20). Morphological variation was higher in *Q. austrocochinchinensis* individuals than in *Q. kerrii* specimens, with standard deviations (σ) for *Q. austrocochinchinensis* and *Q. kerrii* of 0.72 and 0.25 for LR and 0.64 and 0.25 for LAR, respectively.

The putative hybrids of D5 within the hybrid zone had morphological values similar to *Q. kerrii*, with an average of LR = 2.52 (2.22–2.74) and LAR = 0.82 (0.47–1.13). However, the putative hybrids of population C had a sharper leaf apex with LAR = 1.54 (0.86–2.27), although the leaves were as broad as those of *Q. kerrii* with LR = 2.77 (2.35–3.47). In addition, there were seven *Q. austrocochinchinensis* individuals from D3 and D6 with leaf morphological traits similar to those of *Q. kerrii*.

### Population diversity and differentiation

In total, 151 alleles were obtained from the 11 SSR loci used in this study. Some SSRs were highly variable, e.g., CG371, QpZAG16, and QpZAG110, containing more than 20 alleles. These SSRs also had higher Observed Heterozygosity (*H*_O_) and Expected Heterozygosity (*H*_E_) (Table 2) than other loci. Although most frequent alleles were shared by *Q. austrocochinchinensis* and *Q. kerrii*, some loci showed greater variation in allele frequency (Figure S1). Mean Expected Heterozygosity (*H*_E_) across all loci in *Q. austrochichinensis* was 0.706 and higher than that in *Q. kerrii* with 0.595. Species-specific alleles were found at several loci, especially rare alleles restricted to *Q. austrocochinchinensis* (Table 3). Frequency of the private alleles was 34.4 and 12.0% in *Q. austrocochinchinensis* and *Q. kerrii*, respectively. All SSR loci except Qk17611 significantly deviated from HWE (*p* < 0.01). Heterozygote deficiency was found at all loci except Qk17611 and QpZAG36 (*p* < 0.01). The estimation of genetic differentiation between *Q. austrocochinchinensis* and *Q. kerrii* was low across all SSR loci (*F*_ST_ = 0.138). The differentiation among population within species was also low for both species, with *F*_ST_ values of 0.130 and 0.042 for *Q. austrocochinchinensis* and *Q. kerrii*, respectively.

**Table 2:** **Genetic diversity of 11 SSR loci for all individuals of ***Quercus asutrocochinchinensis*** and ***Q. kerrii*****.

**Locus**	**N**	**Na**	**Ne**	**I**	***H*_O_**	***H*_E_**	***uH*_E_**	***F*_IS_**
CG371	96	24	9.352	2.593	0.781	0.893	0.898	0.125
QK20944	101	3	2.610	1.028	0.386	0.617	0.620	0.374
QK17611	69	5	1.615	0.796	0.420	0.381	0.383	−0.104
QK17139	93	11	5.269	1.822	0.688	0.810	0.815	0.151
QK15874	94	17	6.073	2.286	0.617	0.835	0.840	0.261
QpZAG36	93	5	2.884	1.177	0.484	0.653	0.657	0.259
QpZAG16	57	23	15.112	2.894	0.719	0.934	0.942	0.230
QpZAG110	99	28	9.343	2.728	0.677	0.893	0.898	0.242
QpZAG9	96	19	6.958	2.344	0.604	0.856	0.861	0.294
CR627959	93	8	1.612	0.869	0.269	0.380	0.382	0.292
QmC00963	61	8	2.717	1.373	0.459	0.632	0.637	0.274

*N, number or alleles**;** Na, number of different alleles; Ne, number of effective alleles; I, Shannon's Information Index; H_O_, Observed Heterozygosity; H_E_, Expected Heterozygosity; uH_E_, Unbiased Expected Heterozygosity; F_IS_, Fixation Index*.

**Table 3:** **Comparison of genetic diversity and differentiation between ***Quercus austrocochinchinensis*** and ***Q. kerrii*** based on 11 SSR loci**.

	**N**		**An**		**Ap**		***H***_**O**_		***H***_**E**_		***F***_**ST**_		
**Locus**	**QA**	**QK**	**QA**	**QK**	**QA**	**QK**	**QA**	**QK**	**QA**	**QK**	**Between species**	**QA**	**QK**
CG371	54	30	18	17	6	5	0.778	0.800	0.841	0.906	0.041	0.076	−0.001
QK20944	53	33	3	3	0	0	0.302	0.606	0.579	0.501	0.224	0.034	−0.011
QK17611	38	31	5	4	1	0	0.526	0.290	0.467	0.259	0.025	0.027	0.040
QK17139	53	30	9	7	2	0	0.623	0.767	0.770	0.760	0.087	0.185	0.023
QK15874	53	27	15	5	11	1	0.698	0.519	0.892	0.502	0.201	0.009	0.079
QpZAG36	52	29	3	3	0	0	0.385	0.586	0.358	0.439	0.557	−0.002	0.007
QpZAG16	26	22	16	11	10	5	0.615	0.909	0.898	0.875	0.056	−0.123	0.056
QpZAG110	54	31	22	17	8	3	0.611	0.774	0.862	0.887	0.027	0.030	0.070
QpZAG9	50	31	15	15	4	4	0.600	0.677	0.851	0.877	0.004	0.249	0.113
CR627959	51	29	7	3	4	0	0.353	0.172	0.526	0.161	0.076	0.347	0.103
QmC00963	36	11	8	2	6	0	0.500	0.273	0.718	0.351	0.072	0.309	−0.144
All			121	87	52	18	0.545	0.579	0.706	0.593	0.138	0.130	0.042

*QA, two populations of Q. austrocochinchinensis; QK, three populations of Q. kerrii; N, The number of individuals having the allele in each species; An, number of alleles over all the populations for each species; Ap, number of private alleles; H_O_, observed heterozygosity; H_E_, expected heterozygosity; F_ST_, differentiation between populations (species)*.

Application of the seven AFLP primer pairs to 108 individuals resulted in 859 loci, of which 781 were polymorphic. The levels of diversity within each species, either at the population (*H*_W_) or the whole sample level (*H*_t_), were very similar (Table 4). Genetic differentiation between *Q. austrocochinchinensis* and *Q. kerrii* across the 781 markers was low (*F*_ST_ = 0.095, *p* < 0.01) (Table 4). Similarly, the differentiation among populations within species was also low, with *F*_ST_ = 0.0246 (*p* < 0.01) for *Q. austrocochinchinensis* and *F*_ST_ = 0.0667 (*p* < 0.01) for *Q. kerrii*. The *F*_ST_ distribution of 781 AFLP alleles followed an L-shaped curve, which suggested that most of the alleles (471 of 781, 60.3%) had lower *F*_ST_ values (*F*_ST_ < 0.1). Only a few alleles exhibited higher *F*_ST_ values (Figure S2). The *P*_ST_ values for all individuals of the two species, except for the putative hybrids, were larger (0.338) than *F*_ST_ (0.095 for AFLP, 0.082 for SSR). By using BayeScan, 3 out of 781 AFLP loci (0.38%) were identified as outlier loci under directional selection at a threshold of log_10_ PO > 2 (posterior probabilities higher than 0.99) (Figure S3).

**Table 4:** **Comparison of genetic diversity and differentiation between ***Quercus austrocochinchinensi***s and ***Q. kerrii*** based on 781 AFLP loci**.

**Populations**	**N**	***H*_j_**	***H*_t_**	**SE (*H*_t_)**	***H*_W_**	**SE (*H*_W_)**	***F*_ST_**	**Lower 99% *F*_ST_**	**Upper 99% *F*_ST_**
Between species	2	0.2808	0.3084	<0.001	0.0294	0.0025	0.0953	−0.005	0.0096
*Q. austrocochinchinensis*	2	0.2765	0.2847	<0.001	0.2778	0.0015	0.0246	−0.0135	0.0203
*Q. kerrii*	3	0.2815	0.3009	<0.001	0.2808	0.0021	0.0667	−0.0154	0.0156

*N, number of populations; H_t_, total diversity; H_W_, average diversity within population; F_ST_, differentiation between populations*.

### Structure analysis

**Figure 3:**
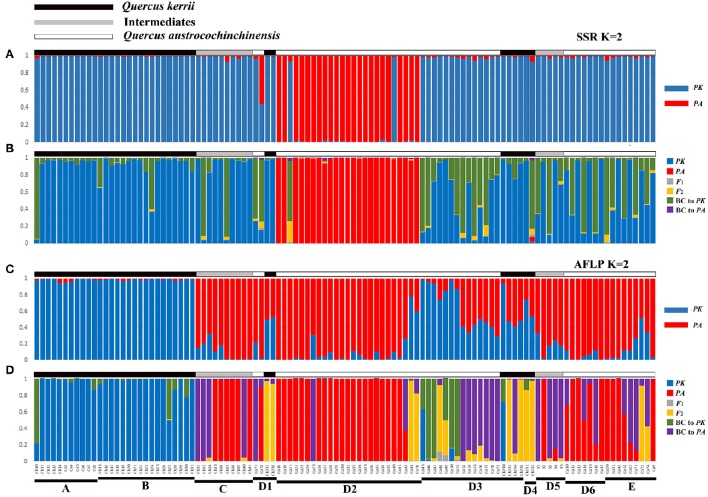
**Genotype class assignment of all 108 individuals of ***Q. austrocochinchinensis***, ***Q. kerrii***, and putative hybrids based on the programs STRUCTURE and NewHybrids using SSR (A,B)** and AFLP **(C,D)** data. Bayesian assignment proportion for K = 2 cluster was determined in STRUCTURE for 11 SSR loci **(A)** and 781 AFLP loci **(C)**. Genotype class assignment using NewHybrids was based on 11 SSR loci **(B)** and 100 AFLP loci with highest *F*_*ST*_**(D)**. There is a one-to-one correspondence among individuals between the four histograms.

The most likely number of clusters (*K*) of STRUCTURE analysis can be inferred by the peak of Δ*K*. We found that *K* = 2 was the optimal *K* value for both AFLP and SSR (Figure S4), representing two species, respectively. Following ten independent STRUCTURE runs with *K* = 2, individuals morphologically identified as *Q. kerrii* were assigned to one cluster with high probability, whereas those morphologically identified as *Q. austrocochinchinensis* were assigned to the other cluster with similarly high probability. Therefore, these two clusters were determined to represent *Q. kerrii* and *Q. austrocochinchinensis*, respectively. The STRUCTURE analysis results of SSR and AFLP are illustrated in Figures 3A,C, respectively, but the results are very different. Among individuals morphologically identified as *Q. kerrii*, both AFLP and SSR recognized populations A and B are *Q. kerrii* purebred population, but the results of the remaining populations are largely contradictory. For example, SSR results assigned most of the individuals in populations/subpopulations C, D1, D3, D4, D5, D6, E, and two individual of D2 to the *Q. kerrii* cluster, and the remaining individuals in D2 to another cluster, regardless of morphological identification. Based on the AFLP results, among the individuals identified as *Q. kerrii* in populations C, D1, D3, and D4, the mean proportion of *Q. kerrii* was 0.575 (0.412–0.941). The two morphological intermediate putative hybrid populations C and D5 contained a high proportion of *Q. austrocochinchinensis* germplasm (with a mean value of 0.901 [0.677–0.992]). Among the individuals identified as *Q. austrocochinchinensis*, in populations D1, D2, and E, the mean proportion of *Q. austrocochinchinensis* germplasm was 89.1% (with the lowest value, 21.7%, found in individual Ca44), but mean proportion was considerably lower in D3 with 34.5% (1.1–71.3%). Given that we considered individuals with *q-*values between 0.6 and 0.9 as backcrosses, we did not identify any backcrossed individuals based on SSRs. However, for AFLP, 25 out of 108 (23.1%) samples were recognized as backcrosses.

The software InStruct (Gao et al., 2007) was used to verify the reliability of SSR results generated by STRUCTURE. The result was highly consistent to the results obtained from STRUCTURE. The optimal *K* value selected by DIC was also 2, similar to the best *K* inferred by STRUCTURE. The D2 subpopulation with pure *Q. austrocochinchinensis* was still easily distinguished from the analysis (Figure S5).

### NewHybrids analysis

Analysis of SSR data using NewHybrids did not obtain results in agreement with the morphological identity of *Q. kerii* and *Q. austrocochinchinensis* individuals examined. Most individuals previously identified as *Q. austrocochinchinensis*, based on morphological characters from subpopulation D2, were still assigned to *Q. austrocochinchinensis*, with high posterior probabilities (>0.974), except for one individual (Ca21). The remaining individuals identified as *Q. austrocochinchinensis* in D1, D3, D6, and E were assigned to either *Q. kerrii* or backcrosses to *Q. kerrii*, as well as the morphological intermediates individuals in Population C and D5. Among the 36 individuals identified as *Q. kerrii*, most were assigned to *Q. kerrii*, with mediate to high probabilities, two (CK10, CK232) were assigned to backcrosses to *Q. kerrii*, with probabilities of 0.935 and 0.802, respectively (Figure 3B).

The assignment of individuals to certain genotypes using AFLP data better fits the morphological identification, but is still very messy. The results obtained from 100, 279, and 450 loci of AFLP were similar in terms of assigning the individuals of *Q. austrocochinchinensis* and *Q. kerrii* from the D region, but the results were different in terms of assigning the individuals in *Q. kerrii* purebred populations A and B, as more backcrosses to *Q. kerrii* were found when adding more loci with low divergence of *F*_ST_ to the analysis (Figure 3, Figures S5B,C). To accurately estimate hybrid classes, alleles with large divergence of *F*_ST_ are required, or misclassification of backcrosses and purebred parental individuals are very likely to happen (Vähä and Primmer, 2006). Therefore, the NewHybrids results obtained from 100 AFLP loci with highest *F*_ST_ value were more reliable and sensitive on assigning the genealogical classes.

Based on morphological features, 57 individuals were identified as *Q. austrocochinchinensis* in populations D and E, of which 30 were assigned to pure *Q. austrocochinchinensis*, one was assigned to F_2_ hybrids, six to backcrosses to *Q. austrocochinchinensis*, and four to backcrosses to *Q. kerrii* with high probabilities. The remaining genotypes were mixtures of certain amounts of *Q. kerrii*, backcrosses to both parental species, and F_2_ (Figure 3D). Among the 15 morphological intermediate individuals of populations C and D5, seven were assigned to *Q. austrocochinchinensis* purebreds, seven to backcrosses to *Q. austrocochinchinensis* with high probabilities, and one was an admixture of *Q. austrocochinchinensis* and its backcrosses. The morphologically identified individuals of *Q. kerrii* in A and B populations were predominantly assigned to pure *Q. kerrii*. The remaining genotypes were mixtures, with few admixtures of certain amounts of *Q. kerrii* and backcrosses to *Q. kerrii*. In D3 and D4, no purebred parental individuals existed, three individuals were assigned to F_2_ with high probability. The genotypes the remaining individuals were either backcrosses to both parental species or a wide spectrum of admixtures of the backcrosses to both parental species, F_1_, F_2_, and *Q. kerrii* purebreds. Of the six individuals of *Q. kerrii* in subpopulations D3 and D4, three were assigned to backcrosses to *Q. austrocochinchinensis*, one to backcross to *Q. kerrii*, and two were identified as F_2_ (Figure 3D).

### Principal coordinate analysis

**Figure 4:**
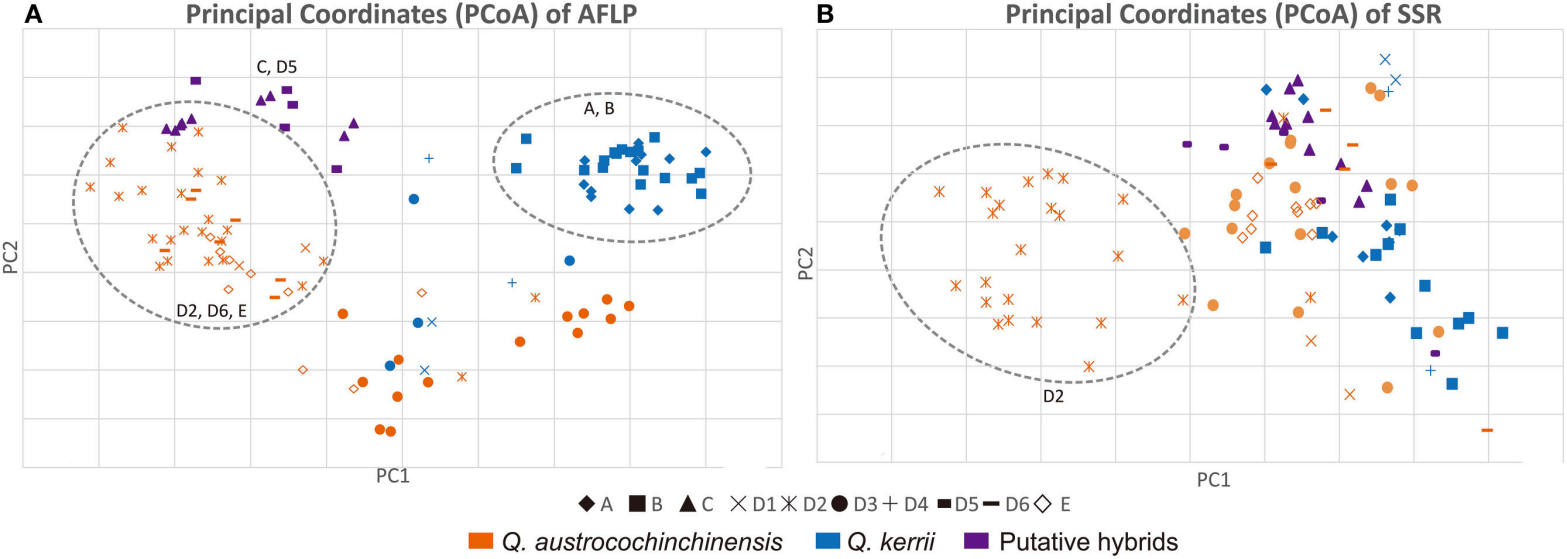
**PCoA plots of AFLP (A)** and SSR **(B)** data. Shapes of the data points on the plot indicate a particular population, as “♦”represents Population A; “■” represents Population B; “▴” represents Population C;“”represents Population E; The rest symbols belongs to Population D, of which “×” represents sub-population D1; “
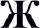
” represents sub-population D2; “•” represents sub-population D3; “+” represents subpopulation D4; “
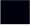
” represents subpopulation D5; “
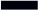
” represents sub-population D6; *Q. austrocochinchinensis, Q. kerrii* and morphological intermediates are represented by orange, blue, and purple, respectively. Clearly clustered individuals are shown within a dashed line circle.

The PCoA results of the AFLP and the SSR data are shown in Figures 4A,B, respectively. The PCoA results were in agreement with the results of the STRUCTURE analysis. Individuals of the two species were mostly separated for both AFLP and SSR data, which indicated that interspecific differentiation was stronger than intraspecific differentiation.

For the AFLP data, most *Q. austrocochinchensis* individuals in D2, D6, and E showed little mixing with *Q. kerrii* and were grouped together (left dashed line circle in Figure 4A). The *Q. kerrii* purebred populations A and B were grouped unambiguously (right dashed line circle in Figure 4A). Populations D1 and D3, which contained both species, were grouped between the two dashed line circles that represented each species. The morphological intermediates in C and D5 were grouped together in the middle position and biased toward *Q. austrocochinchensis*. For SSR analysis, most *Q. austrocochinchinensis* individuals in D2 were grouped together with smaller PC1 values (dashed line circle in Figure 4B). Other samples did not separate into distinct clusters. Still, the result of the PCoA seems to be more reliable, as it revealed more groups than that inferred by STRUCTURE, which only recognized two groups. Except for D2, the putative pure parental species and hybrids were mostly grouped together, but the resolution was not as high as the AFLP data.

## Discussion

### Hybridization and introgression between *Q. kerrii* and *Q. austrocochinchinensis*

*Quercus austrocochinchinensis* is a rare species with only two known distribution sites in China: Xi-Shuang-Ban-Na (XSBN) Nature Reserve in Yunnan province and Ba-Wang-Ling (BWL) Nature Reserve in Hainan Province (Huang et al., 1999; Song et al., 2015). Trees with intermediate morphological form between the two parental species have been found previously in XSBN, suggesting ongoing natural hybridization (Song et al., 2015). However, morphological intermediacy is not invariably associated with hybrids, as it can be a result of hybridization or phenotypic plasticity of the species in these areas (Rieseberg et al., 1993). Therefore, one major objective of this study is to confirm the possible hybridization and introgression in the two species.

Overall, our study revealed that there were fewer *Q. austrocochinchinensis* purebreds than previously expected based on morphological diagnostic traits. Although AFLP and SSR have different distinguishing power on the genotypes of examined samples, both markers revealed the presence of hybridization and introgression between the two species. The SSR data indicated that subpopulation D2 of *Q. austrocochinchinensis* has a unique germplasm composition and might be the only existing purebred. Further STRUCTURE analysis identified that only one individual (Ca78), located at contact zone subpopulation D1, is F_1_. NewHybrids analysis is more sensitive to assign the genotype to different genetic categories and detected a general presence of backcrosses to *Q. kerrii*, both in morphologically identified *Q. kerrii* and *Q. austrocochinchinensis* and the intermediate populations, but no F_1_, F_2_, or backcrosses to *Q. austrocochinchinensis*. The SSR results indicate unidirectional introgression from *Q. kerrii* to *Q. austrocochinchinensis*.

For the AFLP data set, both Bayesian clustering approaches used (implemented in STRUCTURE and NewHybrids) detected an unexpected high number of backcrosses and hybrid genotypes. The threshold values and loci used on assigning individuals to different genetic categories are different in STRUCTURE and NewHybrids. Therefore, the two methods provided different percentages on F_1_ and F_2_ hybrids and backcrosses. STRUCTURE inferred the existence of F_1_ hybrids and backcrosses, whereas NewHybrids inferred absences of F_1_, but predominant backcrosses. STURECTURE is more efficient to evaluate the presence of hybrids in wild populations, whereas NewHybrids algorithm explicitly searches for hybrid and parental classes with assumption of two parental classes, which generally showed higher assignments accuracy than STRUCTURE (Marie et al., 2011). The NewHybrids result suggested that the formation of first-generation hybrids is less likely to occur than the interbreeding of hybrids with purebreds or with other hybrids. The occurrence of F_2_ hybrids and the predominance of first-generation backcrosses to both parental species also reflect recent hybridization between the two species. Meanwhile, the genotypes of some individuals fell between F_2_ hybrids, the first-generation backcrosses, pure *Q. kerrii*, and pure *Q. austrocochinchinensis*, with NewHybrids having no category available to assign them. Those individuals might in reality be second- or later-generation backcrosses or hybrids. This phenomenon is quite prominent in subpopulations D3 and D4, as no purebred individuals exist and the individuals are all hybrids, with varying percentages of backcrossing and parental types. Such evidence indicates that the two locations (D3 and D4) contain historical and ongoing gene flow between the two species.

The intermediate individuals in populations C and D5 are morphologically similar to *Q. kerrii*, mainly in terms of the shallow cupule, persistent trichomes on the leaf abaxial surface, and a relatively thick bark. However, their sharp leaf apex and leaf margin teeth more resemble *Q. austrochochinensis* (Song et al., 2015). The STRUCTURE results of the AFLP and SSR data on individuals of C and D5 were contradictory. The AFLP result indicated that individuals of C and D5 were *Q. austrocochinchinensis* purebred and backcrosses to *Q. austrocochinchinensis*, but the SSR suggested that all individuals in C and D5 were *Q. kerrii* purebreds. In the PCoA analysis, morphologically intermediate individuals from C and D5 were grouped together and located between two parental purebreds (Figure 4). Interestingly, C and D5 are geographically distant. Population D5 is located between regions with *Q. kerrii* and *Q. austrocochinchinensis*, but population C does not have any individuals with the typical features of *Q. austrocochinchinensis*; in addition, all the individuals are young trees, as the area is almost entirely occupied by farming land. The AFLP data revealed a high percentage germplasm of *Q. austrocochinchinensis* in C and D5. A similar situation was also found in population E and subpopulations D1 and D6, indicating that they are genetically “swamped” and that this “swamping” occurred recently, as a high percentage of germplasms of *Q. austrocochinchinensis* can still be detected.

A hybrid swarm is often characterized by a wide spectrum of phenotypic variation, the existence of backcrosses, and high genetic variation (Cockayne and Allan, 1926; Keim et al., 1989). Gathering all the evidences from molecular markers and morphology, there is incidence that a hybrid swarm had been established at the contact region of *Q. kerrii* and *Q. austrocochinchinensis* in XSBN Nature Reserve, especially at populations D3 and D4. The trees of *Q. austrocochinchinensis* in the adjacent regions (locations C, D1, D3–D6, and parts of D2) might have been already genetically swamped. The same situation might also have occurred in population E in Hainan, as SSR indicated that no *Q. austrocochinchinensis* purebreds exist, and the AFLP inferred co-existence of *Q. austrocochinchinensis* purebreds and its backcrosses.

It is worth noting that NewHybrids detected that the genotypes of few individuals contain different admixture levels of backcrosses to *Q. kerrii* and *Q. kerrii* purebreds in the two *Q. kerrii* purebred populations A and B (e.g., CK10, CK27, and CK30), which are distant from all the known populations of *Q. austrocochinchinensis*. Interspecific gene flow is a widespread and ongoing process among oaks, especially in species with close genetic relationship (Coart et al., 2002; Burgarella et al., 2009; Lepais et al., 2009; Moran et al., 2012). There is strong evidence that Neogene climatic changes had little impact on plant distribution in tropical and subtropical Asia (An, 2000; Su et al., 2013; Jacques et al., 2014), although evergreen oaks and other Fagaceae still experienced range shifts in this region (Xu et al., 2015; Jiang et al., 2016; Sun et al., 2016). A recent biogeographical study has indicated that the tropical zone could have extended further north in the geological past than it does today, e.g., the line 20°30′N was the northern biogeographical boundary of the tropical zone in south and southeastern China during the mid-Holocene (Zhu, 2013). The germplasm of *Q. austrocochinchinensis* recovered in *Q. kerrii* purebred populations probably reflects the historical gene flow between the two species and a once wider distribution of *Q. austrocochinchinensis* at this geological time in Indo-China. Future work will need to include more populations of both species, using both maternal markers and high throughput SNP markers to explore the genetic structure and couple the niche modeling to estimate the historical population size; such an approach could provide a better understanding on the genetic patterns of the two oak species.

### Ecological preference

Previous leaf anatomical work has suggested that population E is a *Q. austrocochinchinensis* purebred population with very distinct features compared to *Q. kerrii*, such as narrow leaves, sharp leaf apex, and margin tips (Song et al., 2015). However, the AFLP data showed evidences of hybridization in population E. Across all surveyed populations, only D2 was identified as a pure *Q. austrocochinchinensis* subpopulation by both molecular markers. It is situated in the core region of the Xi-Shan-Ban-Na Nature Reserve with thick woods and geographically isolated from the *Q. kerrii* population, which likely reduces the chances of gene flow and hybridization. Population D2 has a much higher forest canopy density than that of other populations, including population E.

According to our field observation, *Q. austrocochinchinensis* and *Q. kerrii* have different habitat preferences, as the former is likely to grow in closed and moist forests, but the latter tends to grow on open slopes or by roadsides (Figure S6). The fine-scale closed and shady habitats with high humidity might favor the growth of *Q. austrocochinchinensis*, but are also crucial to maintain its genetic distinction. Subpopulations D3, D4, and D5 are located in the buffer area of XSBN Nature Reserve, where human activities are intensified. These regions were inferred as the location of the hybrid swarm of the two species based on our study. Opening habitat as a result of deforestation will likely favor colonization by *Q. kerrii* and facilitate pollen invasion. Our study also showed that introgression of the two oaks occurred at disturbed habitats, and pure *Q. austrocochinchinensis* was found at core protection areas with minimum disturbance. Habitat selection is intimately tied to niche differentiation and coexistence in plant communities (Bazzaz, 1991). Although there are no studies demonstrating the habitat preference of these two species, our data suggests that fine-scale heterogeneous habitats may play an important role in shaping the genetic structure of the two species in areas where they co-exist.

### Discrepancies between AFLP and SSR data

The results of STRUCTURE and NewHybrids showed discrepancies between AFLP and SSR markers (Figure 3). For many samples, AFLP and SSR provided different classifications of species from the same set of samples. Such similar results have also been detected in other plant groups, e.g., *Abies ziyuanensis* (Tang et al., 2008) and the arctic-alpine genus *Draba* (Skrede et al., 2009). This is probably caused by the limited genetic differentiation between the two species, especially in a fine sampling scale. If the two species are not fully differentiated, they will have a number of incompletely differentiated alleles, which may reduce the diagnostic power to distinguish one species from another. In the cases of species with limited genetic differentiation, sampling large numbers of loci across the genome is required when using a molecular approach. The AFLP method has advantages over the SSR method in sampling loci numbers; consequently, AFLP generally reveals higher polymorphism than SSR does (Varshney et al., 2007; Sun et al., 2008; Skrede et al., 2009); it also has a higher assignment success and solution compared to SSR loci (Zeng et al., 2010). Our results also demonstrated that the AFLP data was more consistent with morphology in STRUCTURE and NewHybrids analysis, and the two species separated much better based on using AFLP in PCoA than by using SSR markers. Therefore, we speculate that the results generated from AFLP are more reliable than those obtained from SSR. Our results also demonstrated that when studying hybridization between two genetically closely related oaks, high throughput markers e.g., AFLP or RAD, are more appropriate.

In another aspect, the discrepancies between the two molecular markers might be due to different selective pressures. Similar to the results found by Scotti-Saintagne et al. (2004), the *F*_*ST*_ values of 781 AFLP alleles fit an L-shaped curve. Most alleles (60.3%) have low *F*_ST_ values (*F*_ST_ < 0.1). The results of BayeScan also suggested that only three loci (0.38%) are potentially under selection among the 10 populations. Therefore, most of the AFLP markers are selectively neutral. However, SSR markers may have been subjected to more selective pressure. Morgante et al. (2002) mentioned that SSR repeats tend to occur at transcribed regions of the genome. Selective pressures acting on coding regions are higher than in non-coding regions. In turn, such pressure may lead to genetic differentiation and as a result, SSR loci may have higher genetic differentiation levels than AFLP. We particularly used SSR loci that had been developed from coding sequences, e.g., Qk15874, Qk17139, Qk17611, Qk20944, and CR627959.

Although SSR provided less information than AFLP, it is still a valuable method to compare different marker types. For a pair of species undergoing hybridization, the impermeable genomic regions that are under high selective pressure often serve as a way to maintain species integrity, and thus, these regions accumulate genetic divergence, whereas the regions with low selective pressure are permeable to introgression and show decreased differentiation (Wu, 2001). Because of the higher selective pressures, SSR is more representative of regions with high species integrity, while AFLP is more likely to reflect the effects of gene flow. It is important to note that SSR identified more *Q. kerrii* than AFLP in the STRUCTURE analysis. Moreover, in the PCoA analysis for AFLP, the morphological intermediate populations C and D5 were clustered with *Q. austrocochinchinensis* purebreds. However, for SSR, they were closer to *Q. kerrii* purebreds instead. As discussed above, we suggest that although the trees in the swarm were influenced by the gene flow of *Q. austrocochinchinensis*, the alleles of *Q. kerrii* were preferred in these hybrids. If this is the case, it indicates the high genetic extinction risk of *Q. austrocochinchinensis*.

### Discrepancies between morphological and molecular approaches

Our study revealed the discrepancies between the results inferred by morphological traits and molecular markers. For example, although morphological intermediates only make up a small proportion of samples in the hybrid zone (15 of 61), the molecular method detected more hybrids than expected. Moreover, individuals with one parental morphological characteristic may be identified as another parental purebred, e.g., a part of trees from population D3. There may be two explanations for these discrepancies. First, trees with clear parental characteristics could in fact be F_1_ hybrids or backcrosses, and the morphological phenotypes may be a result of some of the loci having dominant effects on controlling a certain morphological trait. Moreover, hybrids that are in the second or later generation may return to homozygosis at specific loci and thus resemble one parent only (Stebbins, 1950; Rieseberg et al., 1993). However, this offers only a partial explanation, because of the low chance that all loci controlling morphological diagnostic characteristics return to homozygosis at the same time.

Another possibility of the inconsistency is that the phenotype with one of the parental phenotype may be under positive selection in natural habitats, as described previously (Hipp and Weber, 2008). As discussed above, the two species appear to have different habitat preferences. The two populations with typical *Q. austrocochinchinensis* in appearance were all located in the national nature reserve, where canopy density is usually high. The stellate and fused fascicular trichomes and dense stomata, as well as the thick leaf cuticles are important features in seasonal dry areas with strong sun light (Mediterranean-type) climate (Tattini et al., 2007). The glabrous leaves with sharp, spiny teeth were generally an adaptation trait to humid and shady environments (Sun et al., 2003), which may explain why the trees are distributed in dense canopy areas of the Xi-Shuang-Ban-Na Nature Reserve of Yunnan Province and Ba-Wang-Ling Nature Reserves likely show the phenotypes of typical *Q. austrocochinchinensis*, although introgression is common, as this phenotype might have a selective advantage over the hairy, obtuse toothed phenotype in ravine habitats where the environment is usually humid and shaded. Meanwhile, we also could not rule out the possibility that the morphological variation in *Q. kerrii* might encompass the typical morphology of *Q. austrocochinchinensis*. In this case, the morphological features used to distinguish *Q. austrocochinchinensis* from *Q. kerrii* need to be further clarified. Common garden experiment coupling the high throughput genotyping on the different populations of the two taxa and their hybrids are need to reveal how and why traits evolved in response to nature selection and local adaptation in the future.

### Potential threats to *Q. austrocochinchinensis* and conservation strategies

Our data indicates ongoing and historical introgression in the two *Q. austrocochinchinensis* populations. Although the introgression directions inferred by SSR and AFLP are not the same (as SSR indicated unidirectional introgression from *Q. kerrii* to *Q. austrocochinchinensis*, but bidirectional introgression was inferred by AFLP). BayeScan analysis indicated most AFLP loci were selection neutral, but almost all the SSR loci violated HWE assumption. Therefore, AFLP result is more reliable to infer the gene flow between the two species. Recently simulation model study demonstrated when outercrossing rate between the two parental species is different, hybridization can facilitate invasions of the species with high outcrossing rate, even without enhancing local adaption (Mesgaran et al., 2016). Considering the fast shrinking and degradation of habits favoring *Q. austrocochinchinensis* at middle to low elevation of tropical Asia, the ongoing hybrid swarm and the predominance of *Q. kerrii* at co-occur region, *Q. austrocochinchinensis* faces critical extinction risks both in terms of lost habitat and genetic assimilation. Furthermore, our results indicate that only subpopulation D2 maintains its genetic integrity, as the most pure individuals of *Q. austrocochinchinensis* are still purebreds. The other subpopulations in XSBN Nature Reserve and adjacent areas formed a hybrid swarm. Once introgression is detected in an endangered species, protective intervention measures should be adopted immediately to prevent species integrity loss. Otherwise, all subpopulations in the reserve could become hybrid swarms.

Firstly, *in situ* conservation of species genetic resource can be realized by establishing nature reserves. According to our results, the most pure *Q. austrocochinchinensis* population D2 has the highest forest canopy density and lowest human disturbance. Although it is difficult to prove the correlation between ecological integrity and hybrid degree in this case study, we hypothesize that it is important for *in situ* conservation to maintain the existing ecological balance of the habitat of population D2. To avoid further asymmetrical introgressions, forest landscape restoration is also essential for the hybrid *Q. austrocochinchinensis* populations. Secondly, considering the abundance of *Q. kerrii* and the efficient pollen dispersal abilities of oaks, *ex situ* conservation of pure *Q. austrocochinchinensis* should also be considered. Although we cannot claim that *Q. austrocochinchinensis* homozygous individuals still exist, molecular markers and leaf morphological features all identified D2 as the most pure *Q. austrocochinchinensis* population. Therefore, we suggest that the D2 population area should be designated as the priority conservation zone.

## Conclusion

This study suggests that *Q. austrocochinchinensis* in China is experiencing introgression from *Q. kerrii*. The incoherent genetic structure inferred by AFLP and SSR, as well as morphological traits, might be due to the different selective pressures. The extinction risk of *Q. austrocochinchinensis* is higher than previously expected, as much less *Q. austrocochinchinensis* purebreds were detected based on molecular markers; in addition, the species faces ongoing hybrid swarm with *Q. kerrii* and habitat loss in tropical Asia. The subpopulation D2 of *Q. austrocochinchinensis* in the core area of the XSBN Nature Reserve, with unique germplasm and vulnerable to disturbance, should be prioritized for protection. The habitat with high forest canopy density and humidity may act on shaping the genetic structure of the two species at the contact zone. Further studies using high throughput molecular markers and coupling the environmental parameters at fine scale to study the genetic diversity patterns at the co-occurring area of the two species and scan more *Q. kerrii* purebred populations can provide a better understanding on the dynamics of the hybrid zone and determine the underlying genes involved in local adaptation.

## Author contributions

MA and MD designed the study, wrote, and revised the manuscript; MD was responsible for the manuscript submission. SZ and MA performed the experiments; XJ assisted to analyze the data; MD and YS collected the plant materials.

### Conflict of interest statement

The authors declare that the research was conducted in the absence of any commercial or financial relationships that could be construed as a potential conflict of interest.

## Supplementary material

Figure S1 **Allele distributions in ***Q. austrocochinchinensis*** and ***Q. kerrii*** at 11 target loci. (A–K)** Allele frequency of 11 SSR loci. Alleles are arranged on the x-axis and allele frequencies on the y-axis. Blue bar indicates *Q. austrocochinchinensis*; Orange bar indicates *Q. kerrii*. **(A)** Locus GB371; **(B)** locus QK20944; **(C)** locus QK17611; **(D)** locus QK17139; **(E)** locus QK15874; **(F)** QpZAG36; **(G)** locus QpZAG16; **(H)** locus QpZAG110; **(I)** locus QpZAG9; **(J)** locus CR627959; **(K)** locus QmC00963. **(L)** Mean allelic patterns between *Q. austrocochinchinensis* and *Q. kerrii*. **Na**, Number of different alleles; **Na Freq**. ≥ **5%**, number of different alleles with a frequency ≥ 5%; **Ne**, number of effective alleles; **I**, Shannon's Information Index; No. Private Alleles, number of alleles unique to *Q. austrocochinchinensis* and *Q. kerrii*; **No. LComm Alleles** (≤ **25%**), Number of locally common alleles (freq. ≥ 5%) found in 25% or fewer Populations; **No. LComm Alleles** (≤ **50%**), number of locally common alleles (freq. ≥ 5%) found in 50% or fewer populations; ***H***_E_, Expected Heterozygosity; **u*H***_E_, unbiased expected heterozygosity.

Figure S2 *****F***_**ST**_ value distribution of AFLP and SSR loci between ***Q. austrocochinchinensis*** and ***Q. kerrii*****. The x-axis represents the *F*_ST_ values and the y-axis represents the number of loci.

Figure S3 **BayeScan plots of 781 AFLP loci in 10 sampled populations of ***Q. austrocochinchinensis*** and ***Q. kerrii*****. The vertical read line is the threshold (Log_10_(PO) = 2) used for identifying outlier loci. Dots that fall to the right of the threshold line are identified as outlier loci.

Figure S4 **Changes of Δ***K*** from each ***K*** cluster in program STRUCTURE**. Δ*K* is used to identify the most likely number of clusters. For the AFLP and SSR data, *K* = 2 was the most likely *K* value.

Figure S5 **Genotype class assignment of all 108 individuals of ***Q. austrocochinchinensis***, ***Q. kerrii***, and putative hybrids based on the programs InStruct and NewHybrids using SSR (A)** and AFLP **(B,C)** data. *K* = 2 cluster was determined in InStruct for 11 SSR loci **(A)**. The 249 **(B)** and 450 **(C)** AFLP loci with highest *F*_*ST*_ were analyzed, respectively, using NewHybrids.

Figure S6 **Habitat preference of ***Q. austrocochinchinensis*** and ***Q. kerrii*****. *Q. austrocochinchinensis* tends to grow in closed and moist habitat **(A)**, while *Q. kerrii* prefers open and dry habitat **(B)**.

## References

[B1] An M. Deng M. Zheng S. S. Song Y. G. (2016). *De novo* transcriptome assembly and development of SSR markers of oaks *Quercus austrocochinchinensis* and *Q. kerrii* (Fagaceae). Tree Genet. Genomes 12, 103 10.1007/s11295-016-1060-5

[B2] An Z. S. (2000). The history and variability of the East Asian paleomonsoon climate. Quat. Sci. Rev. 19, 171–187. 10.1016/S0277-3791(99)00060-8

[B3] Anderson E. C. (2008). Bayesian inference of species hybrids using multilocus dominant genetic markers. *Philos*. Trans. R. Soc. B Biol. Sci. 363, 2841–2850. 10.1098/rstb.2008.0043 PMC2606736 18508754

[B4] Anderson E. C. Thompson E. A. (2002). A model-based method for identifying species hybrids using multilocus genetic data. Genetics 160, 1217–1229. 11901135 10.1093/genetics/160.3.1217 PMC1462008

[B5] Arnold M. L. (1992). Natural hybridization as an evolutionary process. Annu. Rev. Ecol. Syst. 23, 237–261. 10.1146/annurev.es.23.110192.001321

[B6] Barton N. (2001). The role of hybridization in evolution. Mol. Ecol. 10, 551–568. 10.1046/j.1365-294x.2001.01216.x 11298968

[B7] Bazzaz F. A. (1991). Habitat selection in plants. Am. Nat. 137, s116–s130. 10.1086/285142

[B8] Briggs D. Walters S. M. (1997). Plant Variation and Evolution. Cambridge: Cambridge University Press.

[B9] Burgarella C. Lorenzo Z. Jabbour-Zahab R. Lumaret R. Guichoux E. Petit R. J.. (2009). Detection of hybrids in nature: application to oaks (*Quercus suber* and *Q. ilex*). Heredity 102, 442–452. 10.1038/hdy.2009.8 19240752

[B10] Burger W. (1975). The species concept in Quercus. Taxon 24, 45–50. 10.2307/1218998

[B11] Cavender-Bares J. Pahlich A. (2009). Moluecular, morphological and ecological niche differentiation of sympatric sister oak species, *Quercus virginiana* and *Q. geminata* (Fagaceae). Am. J. Bot. 96, 1690–1702. 10.3732/ajb.0800315 21622355

[B12] Coart E. Lamote V. De Loose M. Van Bockstaele E. Lootens P. Roldán-Ruiz I. (2002). AFLP markers demonstrate local genetic differentiation between two indigenous oak species [*Quercus robur* L. and *Quercus petraea* (Matt.) Liebl.] in Flemish populations. Theor. Appl. Genet. 105, 431–439. 10.1007/s00122-002-0920-6 12582548

[B13] Cockayne L. Allan H. H. (1926). The naming of wild hybrid swarms. Nature 118, 623–624. 10.1038/118623a0

[B14] Coyne J. A. Orr H. A. (2004). Speciation. Sunderland, MA: Sinauer Associates.

[B15] Craft K. J. Ashley M. V. Koenig W. D. (2002). Limited hybridization between *Quercus lobata* and *Quercus douglasii* (Fagaceae) in a mixed stand in central coastal California. Am. J. Bot. 89, 1792–1798. 10.3732/ajb.89.11.1792 21665607

[B16] Curtu A. L. Gailing O. Finkeldey R. (2007). Evidence for hybridization and introgression within a species-rich oak (*Quercus* spp.) community. BMC. Evol. Bio. 7:e218. 10.1186/1471-2148-7-218 17996115 PMC2244923

[B17] Deng M. (2007). Anatomy, Taxonomy, Distribution & Phylogeny of Quercus subg. Cyclobalanopsis (Oersted) Schneid. (Fagaceae). dissertation/PhD's thesis. Kunming Institute of Botany, Chinese Academy of Sciences, Kunming.

[B18] Deng M. Zhou Z. K. Li Q. S. (2013). Taxonomy and systematics of Quercus subgenus Cyclobalanopsis. International Oaks 24, 48–60.

[B19] Evanno G. Regnaut S. Goudet J. (2005). Detecting the number of clusters of individuals using the software STRUCTURE: a simulation study. Mol. Ecol. 14, 2611–2620. 10.1111/j.1365-294X.2005.02553.x 15969739

[B20] Falush D. Stephens M. Pritchard J. K. (2003). Inference of population structure using multilocus genotype data: linked loci and correlated allele frequencies. Genetics 164, 1567–1587. 12930761 10.1093/genetics/164.4.1567 PMC1462648

[B21] Fischer M. C. Foll M. Excoffier L. Heckel G. (2011). Enhanced AFLP genome scans detect local adaptation in high-altitude populations of a small rodent (*Microtus arvalis*). Mol. Ecol. 20, 1450–1462. 10.1111/j.1365-294X.2011.05015.x 21352386

[B22] Foll M. Gaggiotti O. (2008). A genome-scan method to identify selected loci appropriate for both dominant and codominant markers: a bayesian perspective. Genetics 180, 977–993. 10.1534/genetics.108.092221 18780740 PMC2567396

[B23] Gao H. Williamson S. Bustamante C. D. (2007). A Markov chain Monte Carlo approach for joint inference of population structure and inbreeding rates from multilocus genotype data. Genetics 176, 1635–1651. 10.1534/genetics.107.072371 17483417 PMC1931536

[B24] Govaerts R. Frodin D. G. (1998). World Checklist and Bibliography of Fagales (Betulaceae, Corylaceae, Fagaceae and Ticodendraceae). London: Kew Publishing.

[B25] Grant V. (1981). Plant Speciation. New York, NY: Columbia University Press.

[B26] Harrison R. G. (1993). Hybrid Zones and the Evolutionary Process. Oxford: Oxford University Press.

[B27] Hipp A. L. Weber J. A. (2008). Taxonomy of Hill's oak (*Quercus ellipsoidalis*: Fagaceae): evidence from AFLP data. Syst. Bot. 33, 148–158. 10.1600/036364408783887320

[B28] Huang C. C. Chang Y. T. Bartholomew B. (1999). Fagaceae, in Flora of China, Vol. 4, eds Wu C. Y. Raven P. H. (Beijing; St. Louis, MI: Science Press and Missouri Botanical Garden Press), 380–400.

[B29] Huang J. Ge X. Sun M. (2000). Modified CTAB protocol using a silica matrix for isolation of plant genomic DNA. BioTechniques 28, 432–434. 10723554 10.2144/00283bm08

[B30] Jacques F. M. B. Su T. Spicer R. A. Xing Y. W. Huang Y. J. Zhou Z. K. (2014). Late Miocene southwestern Chinese floristic diversity shaped by the southeastern uplift of the Tibetan Plateau. Paleogeogr. Paleoclimatol. Paleoecol. 411, 208–215. 10.1016/j.palaeo.2014.05.041

[B31] Jeffreys H. (1961). Theory of Probability, 3rd Edn. London: Oxford University Press, 432.

[B32] Jiang X. L. Deng M. Li Y. (2016). Evolutionary history of subtropical evergreen broad-leaved forest in Yunnan Plateau and adjacent areas: an insight from *Quercus schottkyana* (Fagaceae). Tree Genet. Genomes 12, 104 10.1007/s11295-016-1063-2

[B33] Keim P. Paige K. N. Whitham T. G. Lark K. G. (1989). Genetic analysis of an interspecific hybrid swarm of Populus: occurrence of unidirectional introgression. Genetics 123, 557–565. 2574697 10.1093/genetics/123.3.557 PMC1203828

[B34] Kleinschmit J. R. Bacilieri R. Kremer A. Roloff A. (1995). Comparison of morphological and genetic traits of pedunculate oak (*Quercus robur* L.) and sessile oak (*Q. petrea* (Matt.) Liebl.). Silvae. Genet. 44, 256–269.

[B35] Kremer A. Dupouey J. L. Deans J. D. Cottrell J. Csaikl U. Finkeldey R. (2002). Leaf morphological differentiation between *Quercus robur* and *Quercus petraea* is stable across western European mixed oak stands. Ann. For. Sci. 59, 777–787. 10.1051/forest:2002065

[B36] Leinonen T. McCairns R. J. S. O'Hara R. B. Merila J. (2013). QST-FST comparisons: evolutionary and ecological insights from genomic heterogeneity. Nat. Rev. Genet. 14, 179–190. 10.1038/nrg3395 23381120

[B37] Lepais O. Petit R. Guichoux E. Lavabre J. Alberto F. Kremer A.. (2009). Species relative abundance and direction of introgression in oaks. Mol. Ecol. 18, 2228–2242. 10.1111/j.1365-294X.2009.04137.x 19302359

[B38] Levin D. A. Francisco-Ortega J. Jansen R. K. (1996). Hybridization and the extinction of rare plant species. Conserv. Biol. 10, 10–16. 10.1046/j.1523-1739.1996.10010010.x

[B39] Lexer C. Kremer A. Petit R. J. (2006). Shared alleles in sympatric oaks: recurrent gene flow is a more parsimonious explanation than ancestral polymorphism. Mol. Ecol. 15, 2007–2012. 10.1111/j.1365-294X.2006.02896.x 16689915

[B40] López-Caamal A. Tovar-Sánchez E. (2014). Genetic, morphological, and chemical patterns of plant hybridization. Rev. Chil. Hist. Nat. 87, 1–14. 10.1186/s40693-014-0016-0

[B41] Lou Y. Zhou Z. K. (2001). Phytogeography of Quercus subg. Cyclobalanopsis. Acta Bot. Yunnan 23, 1–16.

[B42] Lynch M. Milligan B. G. (1994). Analysis of population genetic structure with RAPD markers. Mol. Ecol. 3, 91–99. 10.1111/j.1365-294X.1994.tb00109.x 8019690

[B43] Mallet J. (2005). Hybridization as an invasion of the genome. Trends Ecol. Evol. 20, 229–237. 10.1016/j.tree.2005.02.010 16701374

[B44] Mallet J. (2007). Hybrid speciation. Nature 446, 279–283. 10.1038/nature05706 17361174

[B45] Marie A. D. Bernatchez L. Garant D. (2011). Empirical assessment of software efficiency and accuracy to detect introgression under variable stocking scenarios in brook charr (*Salvelinus fontinalis*). Conserv. Genet. 12, 1215–1227. 10.1007/s10592-011-0224-y

[B46] Mesgaran M. B. Lewis M. A. Ades P. K. Donohue K. Ohadi S. Li C. J.. (2016). Hybridization can facilitate species invasions, even without enhancing local adaptation. Proc. Natl. Acad. Sci. U.S.A. 113, 10210–10214. 10.1007/s10592-011-0224-y 27601582 PMC5018793

[B47] Moran E. V. Willis J. Clark J. S. (2012). Genetic evidence for hybridization in red oaks (Quercus sect. Lobatae, Fagaceae). Am. J. Bot. 99, 92–100. 10.3732/ajb.1100023 22174334

[B48] Morgante M. Hanafey M. Powell W. (2002). Microsatellites are preferentially associated with nonrepetitive DNA in plant genomes. Nat. Genet. 30, 194–200. 10.1038/ng822 11799393

[B49] Muir G. Fleming C. C. Schlotterer C. (2000). Taxonomy - Species status of hybridizing oaks. Nature 405, 1016–1016. 10.1038/35016640 10890434

[B50] Nei M. (1973). Analysis of gene diversity in subdivided populations. Proc. Natl. Acad. Sci. U.S.A. 70, 3321–3323. 10.1073/pnas.70.12.3321 4519626 PMC427228

[B51] Nei M. Li W. H. (1979). Mathematical model for studying genetic variation in terms of restriction endonucleases. Proc. Natl. Acad. Sci. U.S.A. 76, 5269–5273. 10.1073/pnas.76.10.5269 291943 PMC413122

[B52] Ortego J. Bonal R. (2010). Natural hybridisation between kermes (*Quercus coccifera* L.) and holm oaks (*Q. ilex L*.) revealed by microsatellite markers. Plant Biol. 12, 234–238. 10.1111/j.1438-8677.2009.00244.x 20653907

[B53] Peakall R. Smouse P. E. (2012). GenAlEx 6.5: genetic analysis in Excel. Population genetic software for teaching and research–an update. Bioinformatics 28, 2537–2539. 10.1093/bioinformatics/bts460 22820204 PMC3463245

[B54] Phengklai C. (2006). A synoptic account of the Fagaceae of Thailand. *Thai For*. Bull. 34, 53–175.

[B55] Pritchard J. K. Stephens M. Donnelly P. (2000). Inference of population structure using multilocus genotype data. Genetics 155, 945–959. 10835412 10.1093/genetics/155.2.945 PMC1461096

[B56] Rhymer J. M. Simberloff D. (1996). Extinction by hybridization and introgression. Annu. Rev. Ecol. Syst. 27, 83–109. 10.1146/annurev.ecolsys.27.1.83

[B57] Rieseberg L. H. (1995). The role of hybridization in evolution: old wine in new skins. Am. J. Bot. 82, 944–953. 10.2307/2445981

[B58] Rieseberg L. H. (1997). Hybrid origins of plant species. Annu. Rev. Ecol. Syst. 28, 359–389. 10.1146/annurev.ecolsys.28.1.359

[B59] Rieseberg L. H. Ellstrand N. Arnold M. (1993). What can molecular and morphological markers tell us about plant hybridization? Crit. Rev. Plant Sci. 12, 213–241. 10.1080/07352689309701902

[B60] Rousset F. (2008). Genepop'007: a complete re-implementation of the Genepop software for Windows and Linux. Mol. Ecol. Res. 8, 103–106. 10.1111/j.1471-8286.2007.01931.x 21585727

[B61] Salvini D. Bruschi P. Fineschi S. Grossoni P. Kjaer E. D. Vendramin G. G. (2009). Natural hybridisation between *Quercus petraea* (Matt.) Liebl. and *Quercus pubescens* Willd. within an Italian stand as revealed by microsatellite fingerprinting. Plant. Biol. 11, 758–765. 10.1111/j.1438-8677.2008.00158.x 19689784

[B62] Scotti-Saintagne C. Mariette S. Porth I. Goicoechea P. G. Barreneche T. Bodénès C.. (2004). Genome scanning for interspecific differentiation between two closely related oak species [*Quercus robur* L. and *Q. petraea* (Matt.) Liebl.]. Genetics 168, 1615–1626. 10.1534/genetics.104.026849 15579711 PMC1448783

[B63] Skrede I. Borgen L. Brochmann C. (2009). Genetic structuring in three closely related circumpolar plant species: AFLP versus microsatellite markers and high-arctic versus arctic-alpine distributions. Heredity 102, 293–302. 10.1038/hdy.2008.120 19066622

[B64] Song Y. Deng M. Hipp A. Li Q. J. (2015). Leaf morphological evidence of natural hybridization between two oak species (*Quercus austrocochinchinensis* and *Q. kerrii)* and its implications for conservation management. Eur. J. For. Res. 134, 139–151. 10.1007/s10342-014-0839-x

[B65] Stebbins C. Jr. (1950). Variation and Evolution in Plants. New York, NY: Columbia University Press.

[B66] Steinkellner H. Lexer C. Turetschek E. Glössl J. (1997). Conservation of (GA) n microsatellite loci between Quercus species. Mol. Ecol. 6, 1189–1194. 10.1046/j.1365-294X.1997.00288.x 9154990

[B67] Su T. Jacques F. M. B. Spicer R. A. Liu Y. S. Huang Y. J. Xing Y. W. (2013). Post-Pliocene establishment of the present monsoonal climate in SW China: evidence from the late Pliocene Longmen megaflora. Clim. Past 9, 1911–1920. 10.5194/cp-9-1911-2013

[B68] Sun B. N. Cong P. Yan D. Xie S. (2003). Cuticular structure of two angiosperm fossils in neogene from tengchong, yunnan province and its palaeoenvironmental significance. Acta Palaeont. Sin. 42, 216–222.

[B69] Sun Q. B. Li L. F. Li Y. Wu G. J. Ge X. J. (2008). SSR and AFLP markers reveal low genetic diversity in the biofuel plant *Jatropha curcas* in China. Crop Sci. 48, 1865–1871. 10.2135/cropsci2008.02.0074

[B70] Sun Y. Surget-Groba Y. Gao S. (2016). Divergence maintained by climatic selection despite recurrent gene flow: a case study of *Castanopsis carlesii* (Fagaceae). Mol. Ecol. 25, 4580–4592. 10.1111/mec.13764 27447352

[B71] Tamaki I. Okada M. (2014). Genetic admixing of two evergreen oaks, *Quercus acuta* and *Q. sessilifolia* (subgenus Cyclobalanopsis), is the result of interspecific introgressive hybridization. Tree Genet. Genomes 10, 989–999. 10.1007/s11295-014-0737-x

[B72] Tang S. Dai W. Li M. Zhang Y. Geng Y. Wang L.. (2008). Genetic diversity of relictual and endangered plant *Abies ziyuanensis* (Pinaceae) revealed by AFLP and SSR markers. Genetica 133, 21–30. 10.1007/s10709-007-9178-x 17661154

[B73] Tattini M. Matteini P. Saracini E. Traversi M. L. Giordano C. Agati G. (2007). Morphology and biochemistry of non-glandular trichomes in *Cistus salvifolius* L. leaves growing in extreme habitats of the Mediterranean basin. Plant Biol. 9, 411–419. 10.1055/s-2006-924662 17143807

[B74] Tong X. Xu N. N. Li L. Chen X. Y. (2012). Development and characterization of polymorphic microsatellite markers in *Cyclobalanopsis glauca* (Fagaceae). Am. J. Bot. 99, e120–e122. 10.3732/ajb.1100448 22358045

[B75] Ueno S. Tsumura Y. (2008). Development of ten microsatellite markers for *Quercus mongolica* var. crispula by database mining. Conserv. Genet. 9, 1083–1085. 10.1007/s10592-007-9462-4 18506101

[B76] Ueno S. Taguchi Y. Tsumura Y. (2008). Microsatellite markers derived from *Quercus mongolica* var. crispula (Fagaceae) inner bark expressed sequence tags. Genes Genet. Syst. 83, 179–187. 10.1266/ggs.83.179 18506101

[B77] Vähä J. P. Primmer C. R. (2006). Efficiency of model-based Bayesian methods for detecting hybrid individuals under different hybridization scenarios and with different numbers of loci. Mol. Ecol. 15, 63–72. 10.1111/j.1365-294X.2005.02773.x 16367830

[B78] Valbuena-Carabaña M. González-Martínez S. C. Sork V. L. Collada C. Soto A. Goicoechea P. G.. (2005). Gene flow and hybridisation in a mixed oak forest (*Quercus pyrenaica* Willd. and *Quercus petraea* (Matts.) Liebl.) in central Spain. Heredity 95, 457–465. 10.1038/sj.hdy.6800752 16249802

[B79] Van Oosterhout C. Hutchinson W. F. Wills D. P. M. Shipley P. (2004). Micro-checker: software for identifying and correcting genotyping errors in microsatellite data. Mol. Ecol. Notes 4, 535–538. 10.1111/j.1471-8286.2004.00684.x

[B80] Varshney R. K. Chabane K. Hendre P. S. Aggarwal R. K. Graner A. (2007). Comparative assessment of EST-SSR, EST-SNP and AFLP markers for evaluation of genetic diversity and conservation of genetic resources using wild, cultivated and elite barleys. Plant Sci. 173, 638–649. 10.1016/j.plantsci.2007.08.010

[B81] Vekemans X. Beauwens T. Lemaire M. Roldán-Ruiz I. (2002). Data from amplified fragment length polymorphism (AFLP) markers show indication of size homoplasy and of a relationship between degree of homoplasy and fragment size. Mol. Ecol. 11, 139–151. 10.1046/j.0962-1083.2001.01415.x 11903911

[B82] Vos P. Hogers R. Bleeker M. Reijans M. van de Lee T. Hornes M.. (1995). AFLP: a new technique for DNA fingerprinting. Nucleic Acids Res. 23, 4407–4414. 10.1093/nar/23.21.4407 7501463 PMC307397

[B83] Whitney K. D. Ahern J. R. Campbell L. G. Albert L. P. King M. S. (2010). Patterns of hybridization in plants. Perspect. Plant Ecol. Evol. Syst. 12, 175–182. 10.1016/j.ppees.2010.02.002

[B84] Whittemore A. T. Schaal B. A. (1991). Interspecific gene flow in sympatric oaks. Proc. Natl. Acad. Sci. U.S.A 88, 2540–2544. 10.1073/pnas.88.6.2540 11607170 PMC51268

[B85] Wu C. I. (2001). The genic view of the process of speciation. J. Evol. Biol. 14, 851–865. 10.1046/j.1420-9101.2001.00335.x

[B86] Xu J. Deng M. Jiang X. L. Westwood M. Song Y. G. Turkington R. (2015). Phylogeography of *Quercus glauca* (Fagaceae), a dominant tree of East Asian subtropical evergreen forests, based on three chloroplast DNA interspace sequences. Tree Genet. Genomes 11, 805 10.1007/s11295-014-0805-2

[B87] Zalapa J. E. Brunet J. Guries R. P. (2009). Patterns of hybridization and introgression between invasive *Ulmus pumila* (Ulmaceae) and native *U*. rubra. Am. J. Bot. 96, 1116–1128. 10.3732/ajb.0800334 21628262

[B88] Zeng Y. F. Liao W. J. Petit R. J. Zhang D. Y. (2010). Exploring species limits in two closely related Chinese oaks. PLoS ONE 5:e15529. 10.1371/journal.pone.0015529.g001 21152084 PMC2994836

[B89] Zhivotovsky L. A. (1999). Estimating population structure in diploids with multilocus dominant DNA markers. Mol. Ecol. 8, 907–913. 10.1046/j.1365-294x.1999.00620.x 10434412

[B90] Zhu H. (2013). Geographical elements of seed plants suggest the boundary of the tropical zone in China. Paleogeogr. Paleoclimatol. Paleoecol. 386, 16–22. 10.1016/j.palaeo.2013.04.007

