# Supplementary figures and images for: Introgression Threatens the Genetic Diversity of Quercus austrocochinchinensis (Fagaceae), an Endangered Oak: A Case Inferred by Molecular Markers
Source: Front Plant Sci. 2017 Feb 21;8:229. doi: 10.3389/fpls.2017.00229 (PMC5318416; doi:10.3389/fpls.2017.00229)

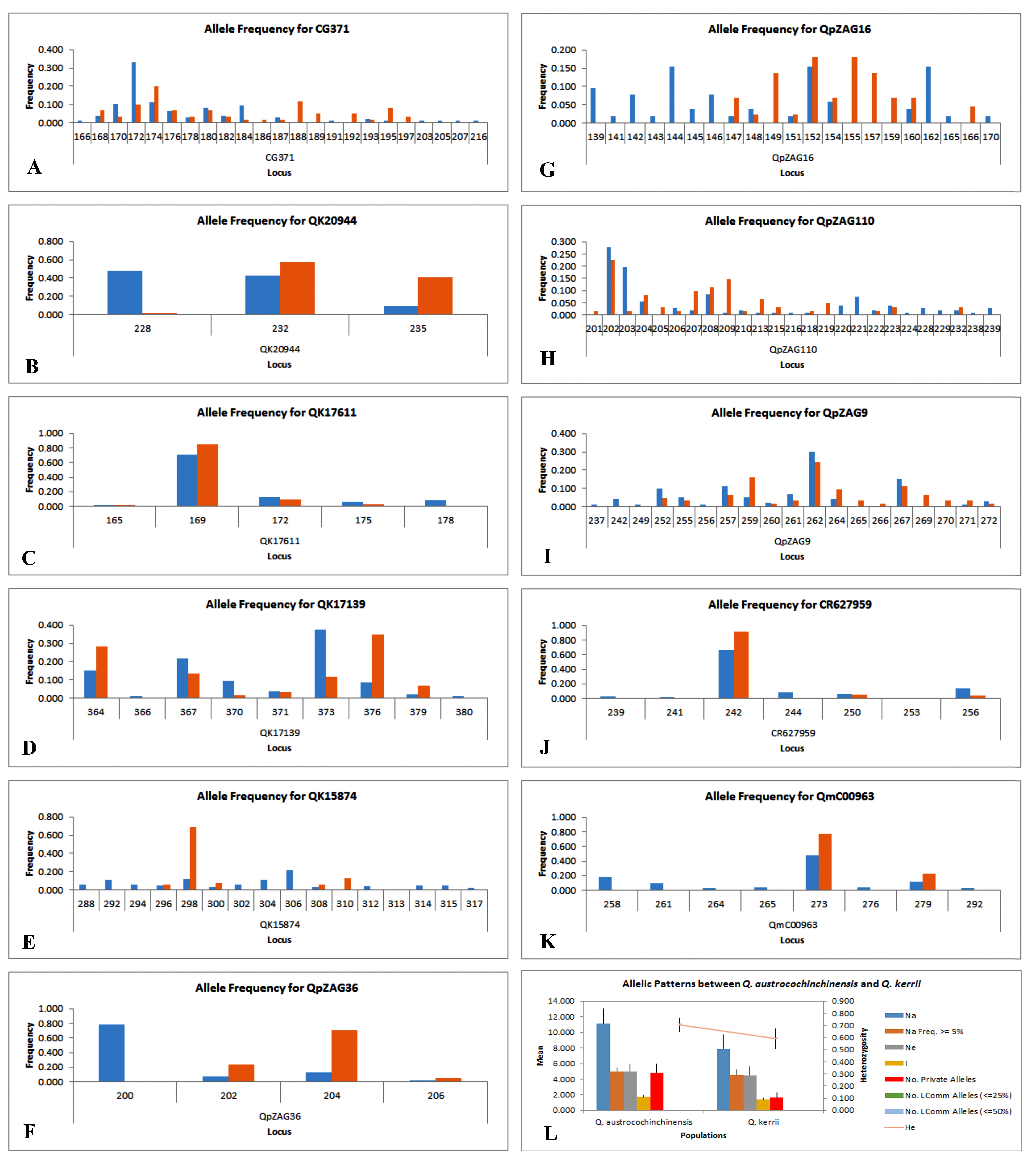

Supplement: Figure S1 — Allele distributions in Q. austrocochinchinensis and Q. kerrii at 11 target loci. (A–K) Allele frequency of 11 SSR loci. Alleles are arranged on the x-axis and allele frequencies on the y-axis. Blue bar indicates Q. austrocochinchinensis; Orange bar indicates Q. kerrii. (A) Locus GB371; (B) locus QK20944; (C) locus QK17611; (D) locus QK17139; (E) locus QK15874; (F) QpZAG36; (G) locus QpZAG16; (H) locus QpZAG110; (I) locus QpZAG9; (J) locus CR627959; (K) locus QmC00963. (L) Mean allelic patterns between Q. austrocochinchinensis and Q. kerrii. Na, Number of different alleles; Na Freq. ≥ 5%, number of different alleles with a frequency ≥ 5%; Ne, number of effective alleles; I, Shannon's Information Index; No. Private Alleles, number of alleles unique to Q. austrocochinchinensis and Q. kerrii; No. LComm Alleles (≤ 25%), Number of locally common alleles (freq. ≥ 5%) found in 25% or fewer Populations; No. LComm Alleles (≤ 50%), number of locally common alleles (freq. ≥ 5%) found in 50% or fewer populations; HE, Expected Heterozygosity; uHE, unbiased expected heterozygosity. [file Image1.JPEG]

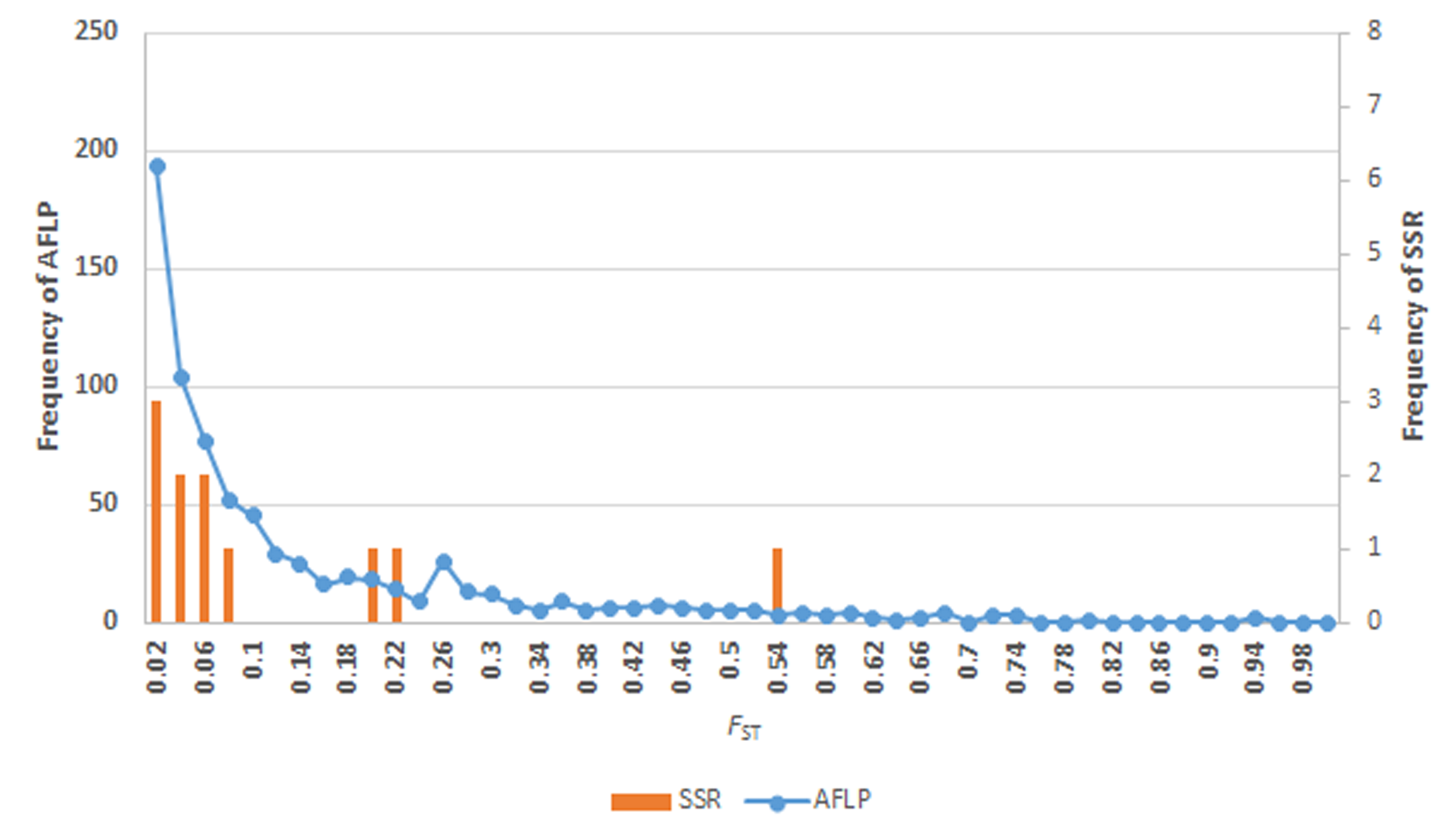

Supplement: Figure S2 — FST value distribution of AFLP and SSR loci between Q. austrocochinchinensis and Q. kerrii. The x-axis represents the FST values and the y-axis represents the number of loci. [file Image2.JPEG]

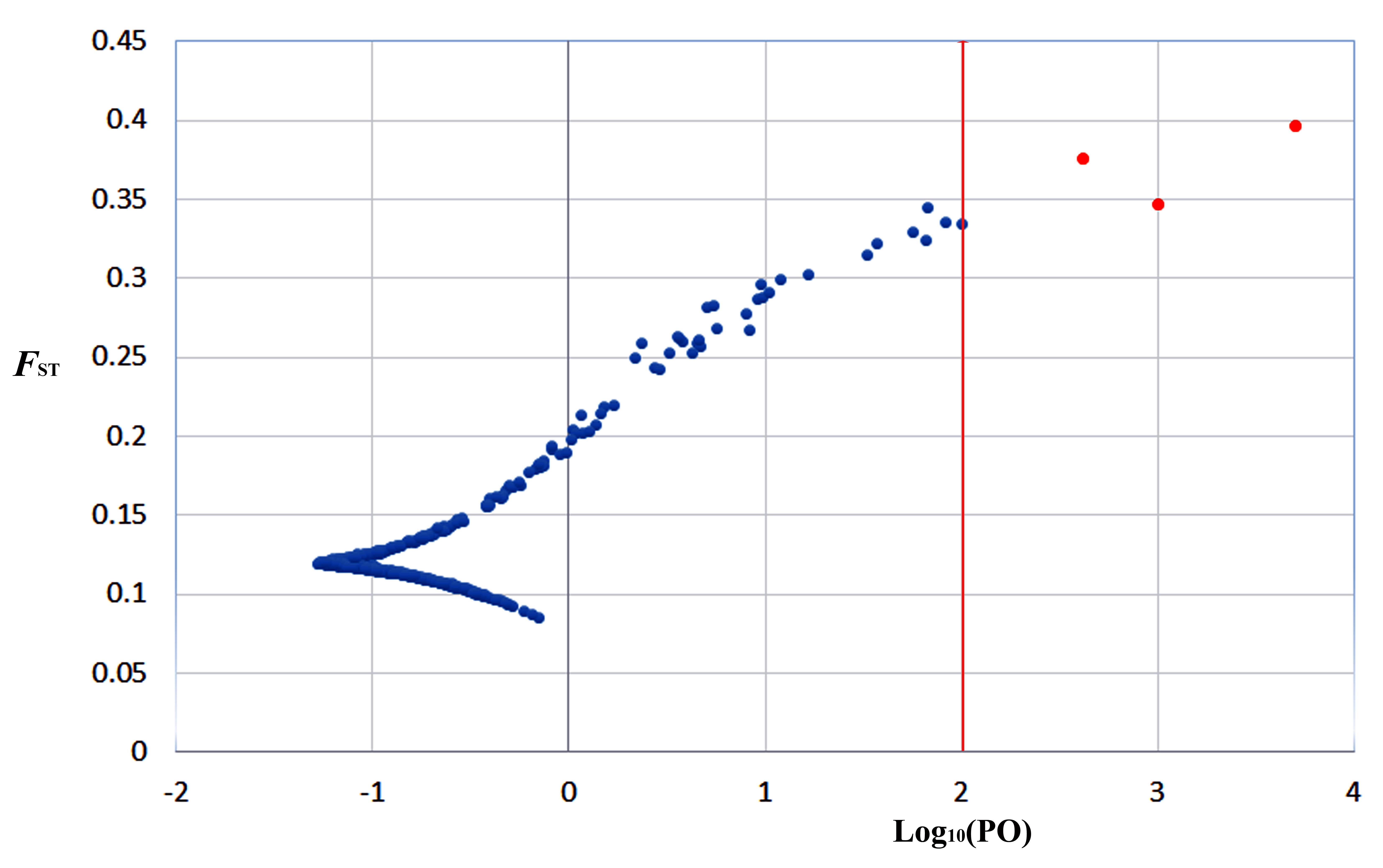

Supplement: Figure S3 — BayeScan plots of 781 AFLP loci in 10 sampled populations of Q. austrocochinchinensis and Q. kerrii. The vertical read line is the threshold (Log10(PO) = 2) used for identifying outlier loci. Dots that fall to the right of the threshold line are identified as outlier loci. [file Image3.JPEG]

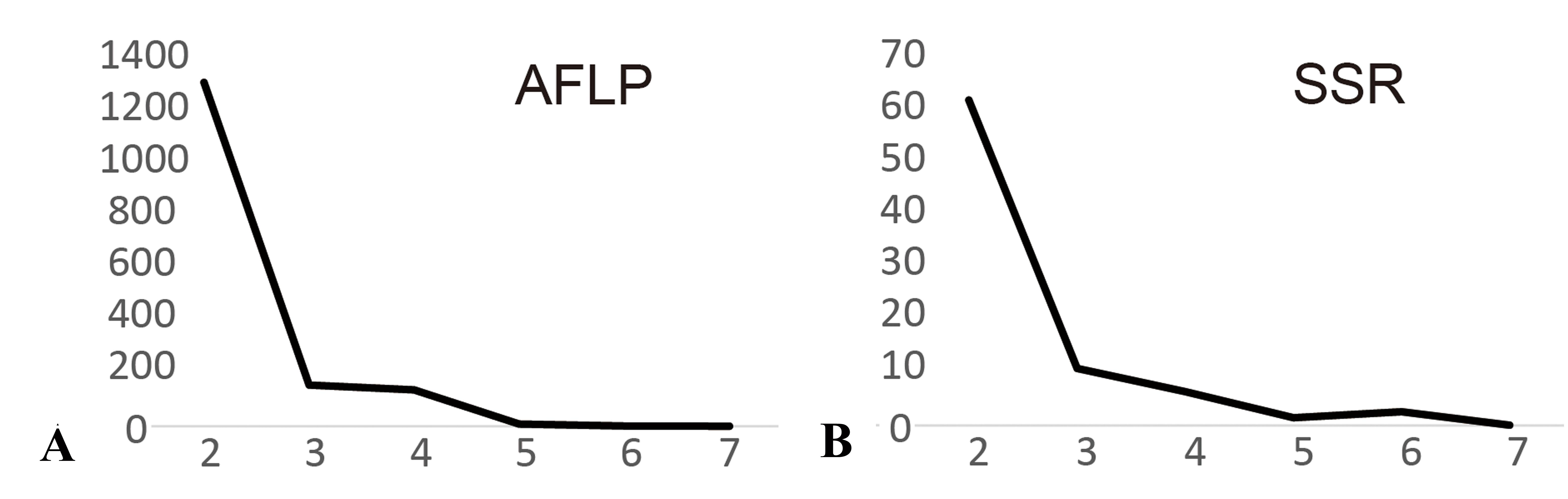

Supplement: Figure S4 — Changes of ΔK from each K cluster in program STRUCTURE. ΔK is used to identify the most likely number of clusters. For the AFLP and SSR data, K = 2 was the most likely K value. [file Image4.JPEG]

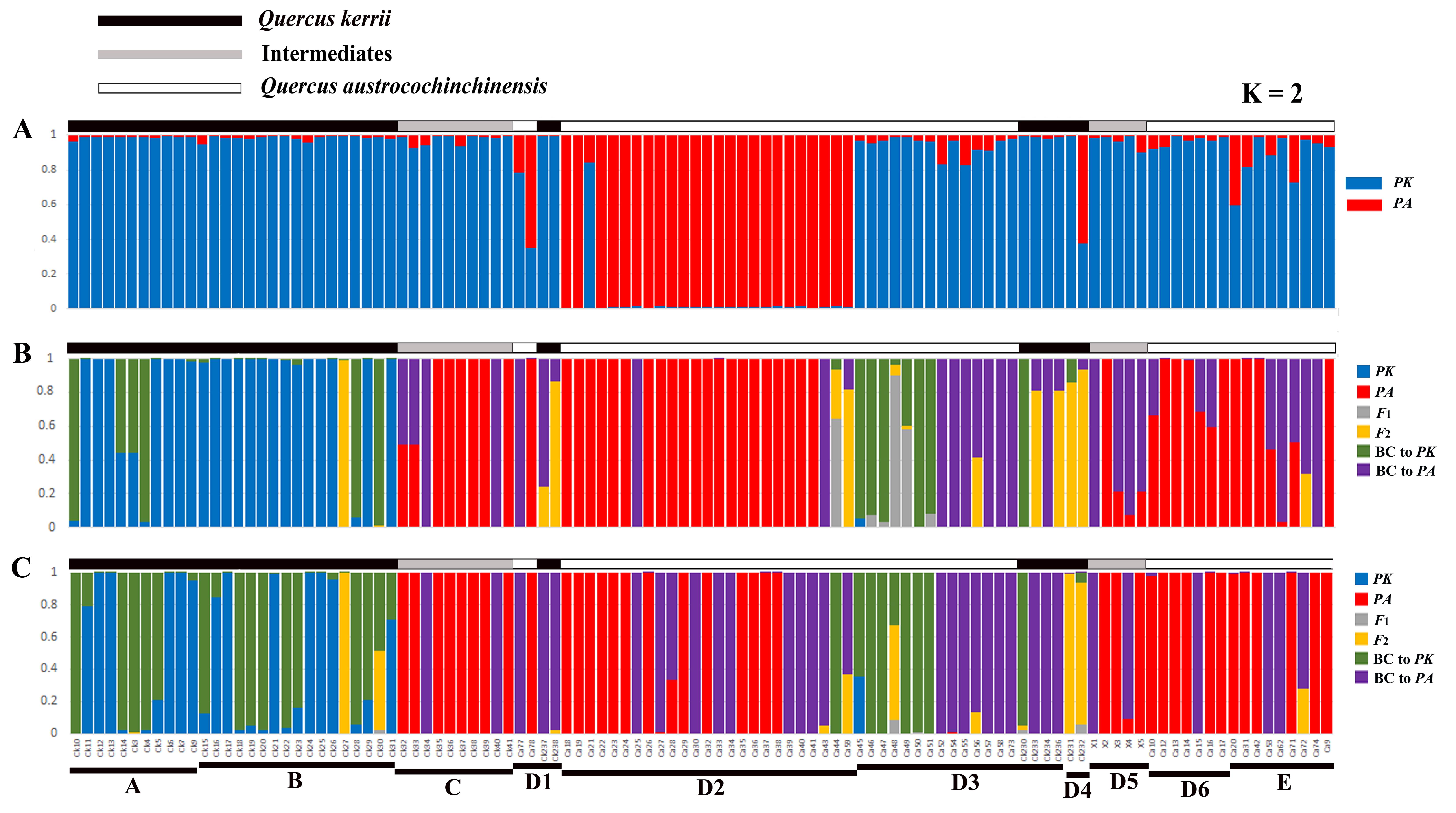

Supplement: Figure S5 — Genotype class assignment of all 108 individuals of Q. austrocochinchinensis, Q. kerrii, and putative hybrids based on the programs InStruct and NewHybrids using SSR (A) and AFLP (B,C) data. K = 2 cluster was determined in InStruct for 11 SSR loci (A). The 249 (B) and 450 (C) AFLP loci with highest FST were analyzed, respectively, using NewHybrids. [file Image5.JPEG]

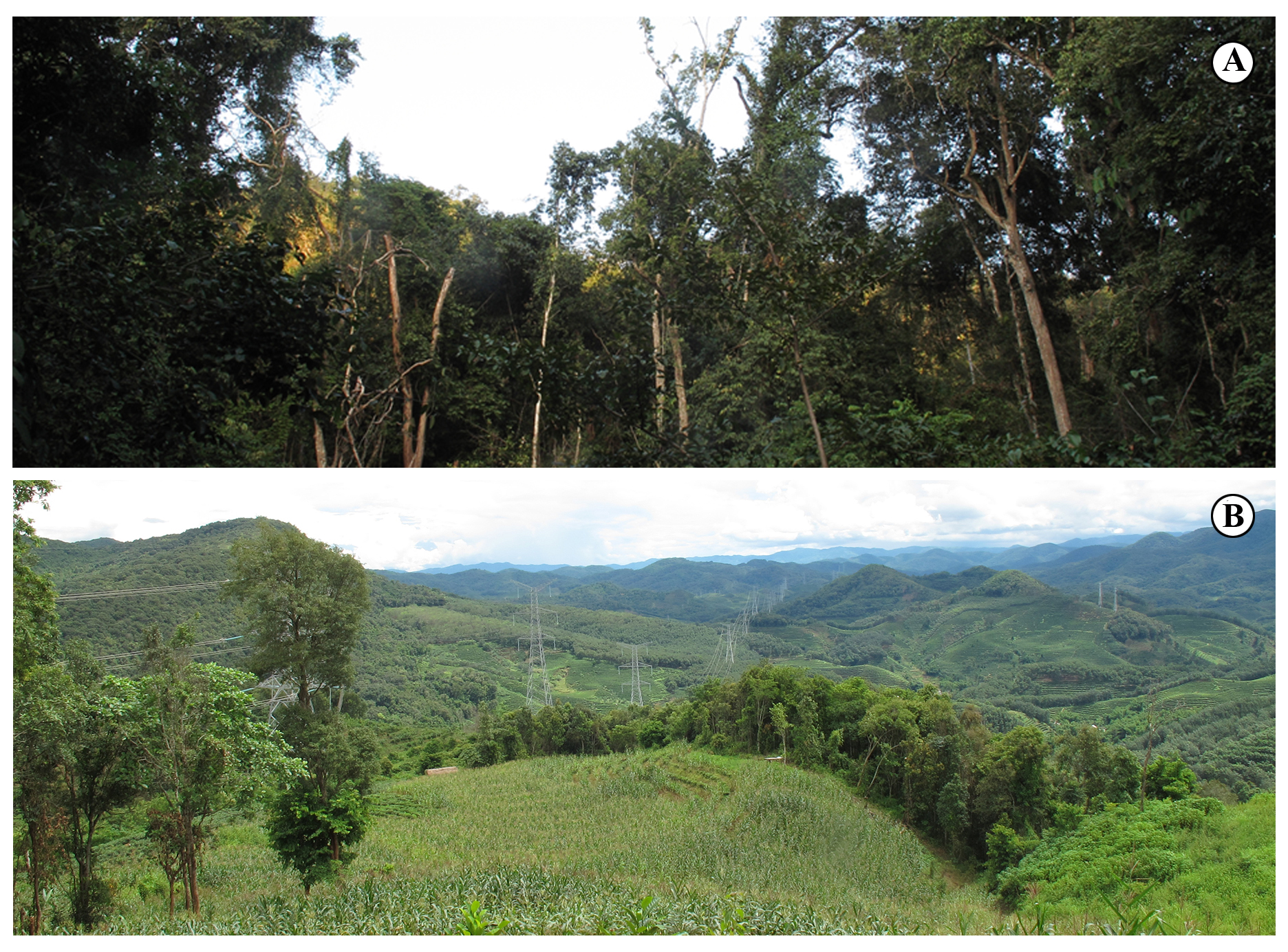

Supplement: Figure S6 — Habitat preference of Q. austrocochinchinensis and Q. kerrii. Q. austrocochinchinensis tends to grow in closed and moist habitat (A), while Q. kerrii prefers open and dry habitat (B). [file Image6.JPEG]
